# Engineering of Green Carbon Dots for Biomedical and Biotechnological Applications

**DOI:** 10.3390/molecules29184508

**Published:** 2024-09-23

**Authors:** Junjie Shang, Qian Zhou, Kehan Wang, Yunlin Wei

**Affiliations:** Faculty of Life Science and Technology, Kunming University of Science and Technology, Kunming 650500, China; sjjnjy117327@google.com (J.S.); 17687026368@163.com (Q.Z.); 18087916582@163.com (K.W.)

**Keywords:** carbon dots, green precursors, green synthesis, optical properties, bio-applications

## Abstract

Carbon dots (CDs) are attracting increasing research attention due to their exceptional attributes, including their biocompatibility, water solubility, minimal toxicity, high photoluminescence, and easy functionalization. Green CDs, derived from natural sources such as fruits and vegetables, present advantages over conventionally produced CDs, such as cost-effectiveness, stability, simplicity, safety, and environmental friendliness. Various methods, including hydrothermal and microwave treatments, are used to synthesize green CDs, which demonstrate strong biocompatibility, stability, and luminescence. These properties give green CDs versatility in their biological applications, such as bioimaging, biosensing, and drug delivery. This review summarizes the prevalent synthesis methods and renewable sources regarding green CDs; examines their optical features; and explores their extensive biological applications, including in bioimaging, biosensing, drug/gene delivery, antimicrobial and antiviral effects, formatting of mathematical components, cancer diagnosis, and pharmaceutical formulations.

## 1. Introduction

**Figure 1:**
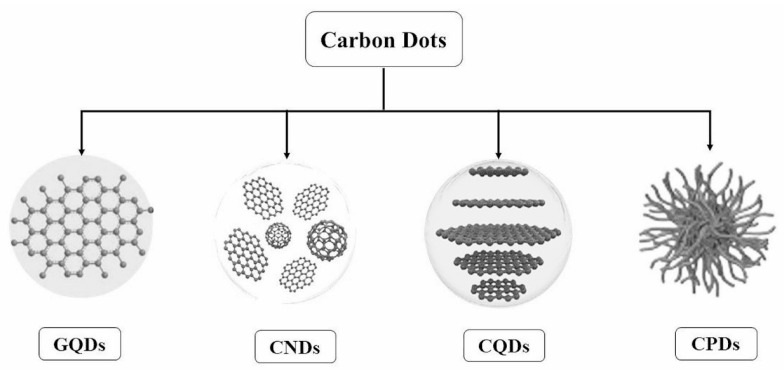
Classification of carbon dots [19].

Various carbon-based nanomaterials, including carbon nanotubes (CNTs) [1], fullerene [2], graphene [3], graphene quantum dots (GQDs) [4], carbon dots (CDs) [5], carbon nanohorns (CNHs) [6], and carbon nano-onions (CNOs) [7], have garnered significant interest for their unique chemical and physical properties. Among these, CDs represent the latest advancement, distinguished by their excellent biocompatibility, water solubility, low toxicity, high photoluminescence quantum yield (QY), and ease of functionalization [8,9,10,11,12,13,14,15,16]. Notably, CDs exhibit advantages over traditional quantum dots (QDs) in biological applications [17,18], underscoring their potential in the field. Based on their properties, CDs are classified into GQDs, carbon quantum dots (CQDs), carbon nanodots (CNDs), and carbonized polymer dots (CPDs), as shown in Figure 1 [19].

Fluorescent nanoparticles, now known as CDs, were first identified, unintentionally, by Xu et al. [20] during the purification of single-walled CNTs (SWCNTs) in 2004. Sun et al. [21] later refined these particles, naming them CDs, which are typically smaller than 10 nm. Historically, CDs have been synthesized from various chemical precursors, like graphite and charcoal target [22,23,24,25], using methods such as arc discharge and laser ablation [26,27]. However, these methods often involve toxic chemicals and high temperatures and are costly and time intensive.

In response, “green CDs” synthesized from natural or renewable sources have gained prominence because of their eco-friendliness, cost-effectiveness, and excellent biocompatibility. These particles are derived from diverse green sources, including fruits (orange [28], lemon [29], and carica papaya juices [30]), vegetables (tomato [31], Chinese yam [32], and onion [33]), spices (purple perilla [34], garlic [35], and coriander leaves [36]), non-edible plants (Azadirachta indica leaves [37], Osmanthus fragrans [38], and ginkgo leaves [39]), animal derivatives (wool [40], silkworm chrysalis [41], and milk [42]), human derivatives [43] (human hair [44], fingernails [45], and urine [46]), micro-organisms (*Bacillus cereus* [47], *Escherichia coli* [48], and *lactic acid bacteria* [49]), waste materials (lychee waste [50], sugarcane bagasse pulp [51], and soybean residuals [52]), herbal medicines [53], beverages [32,54], and bakery products [55,56]. Moreover, small biomolecules, such as vitamins [57,58], proteins [59], amino acids [60], and carbohydrates [61,62], have been utilized in green CDs synthesis, broadening their applications in biomedicine and biotechnology because of their sustainable and benign nature.

Numerous reviews on CDs have detailed their synthesis, properties, and various applications, including in chemical sensors, bioimaging, catalysis, and solar cells [15,63]. However, a notable gap remains in the literature specifically addressing green CDs, especially regarding their biomedical and biotechnological applications. Recent works, such as that by Luo et al. [64], have delved into green synthesis methods and general applications for CDs, but have not extensively covered their biomedical relevance. This review seeks to address this gap by focusing on the applications of green CDs in the biomedical and biotechnological sectors, which are attracting increasing interest. We explore their roles in bioimaging, biosensing, drug and gene delivery, antimicrobial and antiviral activities, photothermal and photodynamic therapies, cancer diagnosis, and drug formulation, highlighting recent advancements and potential future directions.

## 2. Synthesis of Green CDs

Synthetic methodologies for CDs can be broadly categorized into “top–down” and “bottom–up” strategies. Top–down approaches, such as laser ablation, arc discharge, electrochemical oxidation, and ultrasonication, typically involve intense physical or chemical processes. However, the synthesis of green CDs predominantly employs bottom–up methods, including hydrothermal treatment, microwave heating, thermal processing, and extraction. These bottom–up techniques have several advantages—eco-friendliness, low-temperature operation, simplicity, cost-effectiveness, and scalability—aligning with the principles of green chemistry and sustainable production.

### 2.1. Hydrothermal Treatment

Hydrothermal treatment stands out as the preferred method for synthesizing CDs because of its simplicity, cost efficiency, energy efficiency, and environmental friendliness. This process typically involves high-temperature reactions in a stainless steel autoclave. Notably, Prathap et al. [65] utilized biomass, specifically Prosopis juliflora, to produce CDs, marking a significant advancement in green CDs synthesis. This type of CD has found antibacterial and bioimaging applications. Various natural materials, including fruits (e.g., oranges) and vegetables (e.g., cabbage), have been used as sources for green CDs production, demonstrating applications from bioimaging to detecting bioactive molecules, such as dopamine.

**Figure 2:**
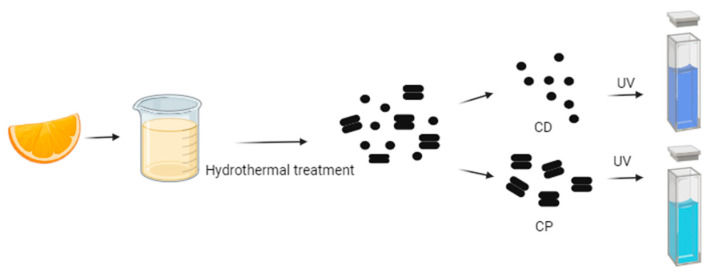
Green CDs synthesized from orange juice emitted bright-blue fluorescence under UV light irradiation, whereas CPs indicated coarse particles with less fluorescence (figure created in BioRender.com; UV—ultraviolet).

**Figure 3:**
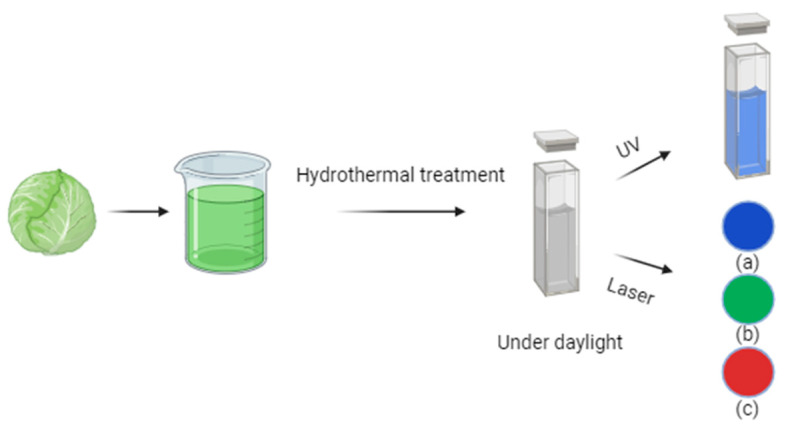
Green CDs synthesized from cabbage showed (a) blue, (b) green, and (c) red emissions under a confocal microscope when excited by 405, 480, and 543 nm lasers, respectively (figure created in BioRender.com).

**Figure 4:**
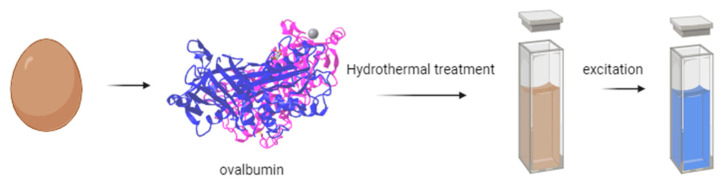
Green CDs synthesized from eggs (figure created in BioRender.com).

For instance, Gholipour et al. [66] synthesized green CDs from orange juice (Figure 2), which served as fluorescent probes for dopamine detection. Similarly, Alam et al. [67] developed CDs from cabbage, which showcased multicolor imaging capabilities in human cells (Figure 3). In another study, CDs were created from hen eggs via hydrothermal treatments on ovalbumin (OVA), exhibiting a quantum yield of 34.5% [68] (Figure 4). Additionally, in vitro imaging studies suggested that the CDs possessed extraordinary optical properties and biocompatibility. Diverse materials, including carrot juice [69], tea waste [65], onions [33], bovine serum albumin [70], coffee ground waste [71], and banana leaves [72], have been explored for CDs synthesis, illustrating the method’s versatility and the wide range of potential applications for these eco-friendly nanoparticles.

### 2.2. Microwave Irradiation

Microwave irradiation synthesis is acclaimed for its rapid, efficient, and cost-effective production of CDs, significantly enhancing their yield and quality compared to other methods. In this method, natural precursors are dissolved in a solvent and subjected to microwave heating, facilitating quick and effective synthesis. For instance, Architha et al. [73] synthesized CDs from Mexican mint leaves using this technique, achieving notable quantum yields and demonstrating their utility in biosensing and cell imaging. In another study, Vu Nu et al. [74] leveraged a similar approach with microalgae to produce CDs that improved the photodegradation capabilities of TiO_2_ nanoparticles compared to pristine TiO_2_ (83% and 27%, respectively). Nkeumaleu et al. [75] offered an eco-friendly solution by synthesizing CDs from recycled microbrewery waste, showcasing their applications in sensing technology for water treatment, food quality, and safety detection. Additionally, human fingernails have been explored as a precursor in microwave-assisted CDs synthesis, producing particles useful in dye sensing and biological imaging [45]. Other sources, such as mango [76], aloe [77], sucrose [78], and Momordica charantia fruits [79], have also been utilized, underscoring the method’s adaptability and the broad scope of potential applications for the synthesized CDs.

### 2.3. Heating

**Figure 5:**
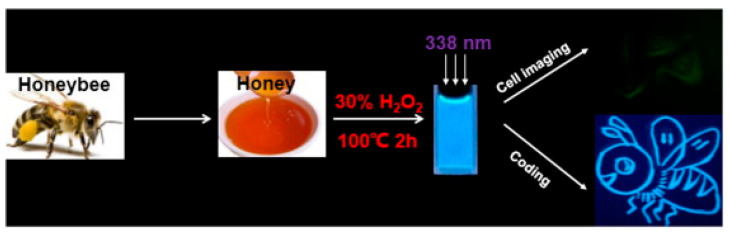
Schematic illustration of synthesis of CDs and cell imaging and coding, whereas three white arrows indicated the maximum excitation spectrum of CDs synthesized is 380 nm [82].

Heating is recognized as a straightforward, eco-friendly, and cost-efficient technique for producing green CDs. Rawat et al. [80] demonstrated this by synthesizing CDs from watermelon juice at 160 °C and further exploring their potential for detecting Pb^2+^ metal ions in polluted water and in a human cervical cancer cell line. Among the various functionally modified CDs, only the synthesized CDs demonstrated excellent selectivity for Pb^2+^ ions. Similarly, Tohamy et al. [81] utilized a basic microwave heating method, with the precursors including urea and various cellulose forms, to produce CDs, showcasing their applicability to environmental monitoring and chemical sensing. Honey [82] was also explored as a precursor for green CDs synthesis via heating, underlining the method’s versatility and the range of natural materials that can be employed. These developments underscore the heating method’s role in advancing green nanotechnology, particularly for applications in the biomedical and environmental sectors, as shown in Figure 5.

### 2.4. Extraction

**Figure 6:**
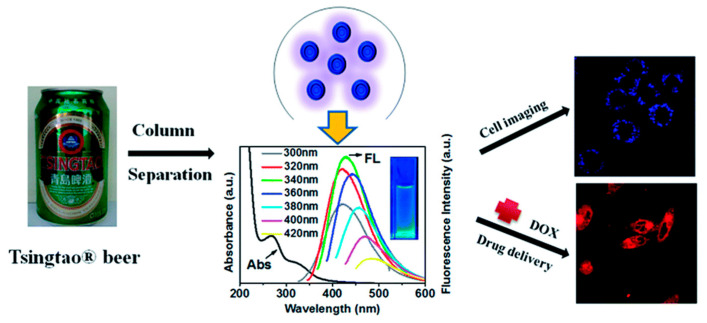
CDs present in beer for breast cancer cell imaging and drug delivery [32].

A cutting-edge approach to green CDs production involves direct extraction from commonly consumed beverages and bakery products, sidestepping conventional synthesis processes. For instance, Wang et al. [32] obtained green CDs from Tsingtao^®^ beer; they first degassed the beer, and then concentrated and filtered it, followed by purification using gel-filtration chromatography. These CDs demonstrated excellent biocompatibility, proving useful in bioimaging applications in cells and fish (Figure 6). Furthermore, the CDs showed potential when applied as nanocarriers in anticancer therapy, owing to the doxorubicin (DOX)-conjugated CDs (DOX-CDs) inducing prolonged cytotoxicity compared to free DOX. Similar extraction methods have been applied to other beverages, such as Coca-Cola [83] and instant coffee [84], among others [85], to derive green CDs. Additionally, the extraction of CDs from thermally processed foods has been explored. For example, Cong et al. [55] successfully isolated green CDs from pizza, assessing their biodistribution and cytotoxicity in various biological models. This novel extraction method has also been extended by other studies using baked lamb [86], barbeque [87], and grilled Spanish mackerel [88], revealing the great potential for sourcing green CDs from everyday foods.

### 2.5. Other Methods

**Figure 7:**
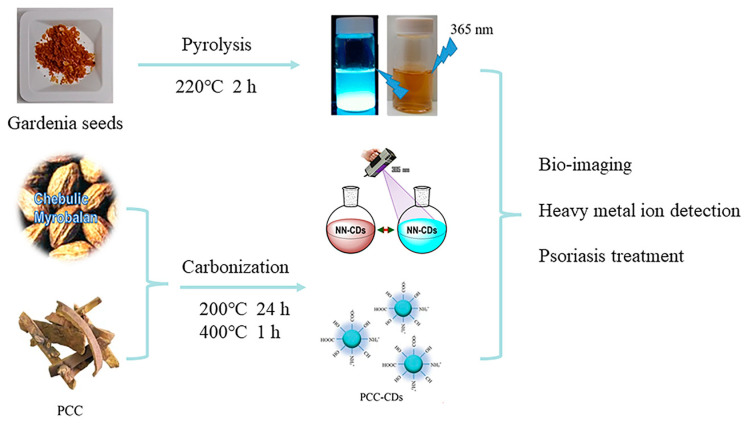
CDs obtained by pyrolysis and carbonization, where NN-CDs indicates natural nitrogen-doped CDs and PCC indicates phellodendri chinensis cortex [89,90,91].

Beyond the traditional synthesis methods, researchers are adopting straightforward and novel techniques with which to produce green CDs (Figure 7). Pyrolysis is one such method, exemplified by Tsai et al. [89], who synthesized green CDs from gardenia seeds using a gentle one-step technique, yielding CDs that served as effective probes for Escherichia coli imaging.

Carbonization offers another eco-friendly synthesis pathway, utilizing organic materials under inert conditions. For instance, Atchudan et al. [90] created CDs from Chebulic Myrobalan through carbonization, yielding water-soluble CDs with stable photoluminescence, ideal for the detection of heavy metal ions in an aqueous medium. Similarly, Zhang et al. [91] adopted a method with phellodendri chinensis cortex, producing CDs with the potential to treat psoriasis in clinical applications.

In Table 1, we summarize the size distribution and maximum yield of all the carbon dots synthesized by the methods cited in this article. Obviously, hydrothermal treatment is the most commonly used method for synthesizing green CDs, favored for its straightforwardness, cost efficiency, and environmental benefits, albeit with the drawback of being time intensive. Meanwhile, microwave irradiation, heating, and extraction are emerging as notable alternatives within the bottom–up strategies, offering advantages in terms of efficiency, economy, and speed. Consequently, these methods present promising avenues for future research and development in the synthesis of green CDs, warranting increased attention due to their potential to optimize and expedite production processes.

## 3. Optical Properties

In this section, we provide an overview of the typical optical properties shared by CDs, regardless of their diverse structural variations. These properties encompass optical absorbance, photoluminescence, and up-conversion fluorescence. Despite the structural diversity among different CDs, these optical characteristics are remarkably consistent, defining the unique and valuable optical behavior of CDs. By summarizing these properties, we aim to highlight the fundamental aspects that contribute to the wide-ranging applicability of CDs in various fields, especially in optical applications and materials science.

### 3.1. Absorbance

Generally, the UV–visible absorption spectra of green CDs are characterized by a prominent peak in the UV region, extending into the visible range. Specifically, absorption peaks between 230 and 270 nm are attributed to the π–π* transitions of C=C bonds, while those in the 300–330 nm range correspond to the *n*–π* transitions of C=O bonds [72,95,96]. Moreover, the absorption features of green CDs can be further modulated by variations in their surface functional groups, indicating how the surface chemistry can impact their optical properties. Understanding these absorption characteristics is crucial for exploring the applications of green CDs, particularly in areas requiring specific optical responses.

### 3.2. Photoluminescence

**Figure 8:**
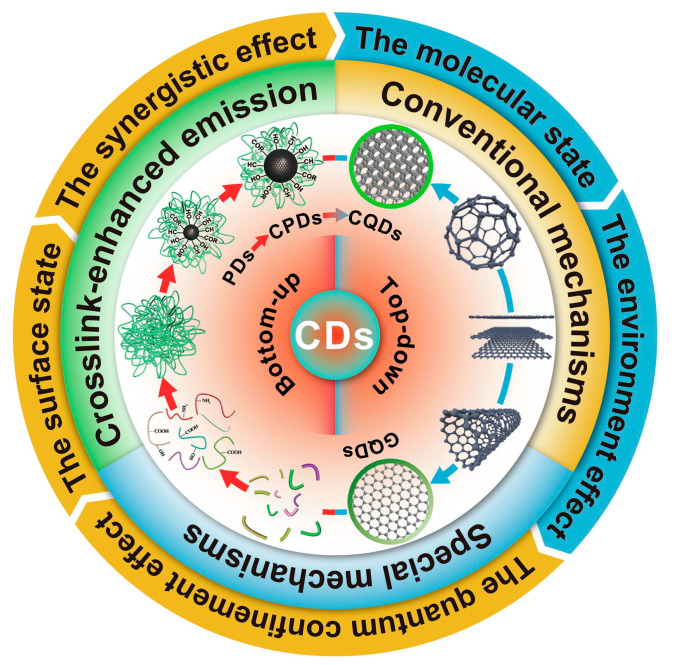
PL mechanisms of CDs, where PDs indicate polymer dots, a kind of CD [98].

The fluorescence spectra of green CDs are notably broad, spanning from the deep-UV to near-infrared regions, with common emissions in the blue-light range. The specific mechanisms behind this broad emission spectrum remain elusive despite extensive study. The proposed explanations for these diverse fluorescence behaviors include the effects related to particle size, surface defects, surface states, element doping, aromaticity, and oxidation levels. The observed excitation-dependent fluorescence, where the emission peaks shift with the excitation wavelength, may be due to CDs’ heterogeneous size distributions, various surface defect states, or distinct emissive sites. Yi et al. [97] proposed a single change in the pH of the synthesis conditions, which had no effect on the CDs’ intrinsic core states and avoided the mutual influence of multiple PL origins. Li et al. [83] suggested that this broad emission spectrum relates to complex excited states or multiple emissive centers. Furthermore, Ai et al. [98] categorized photoluminescence (PL) mechanisms into three types based on the structural influences—internal factors (such as conjugation, surface states, and synergistic effects), external factors (involving molecular states and environmental impacts), and crosslink-enhanced emissions—outlining a multifaceted view of CDs’ photoluminescent properties, as shown in Figure 8.

### 3.3. Up-Conversion Fluorescence

**Figure 9:**
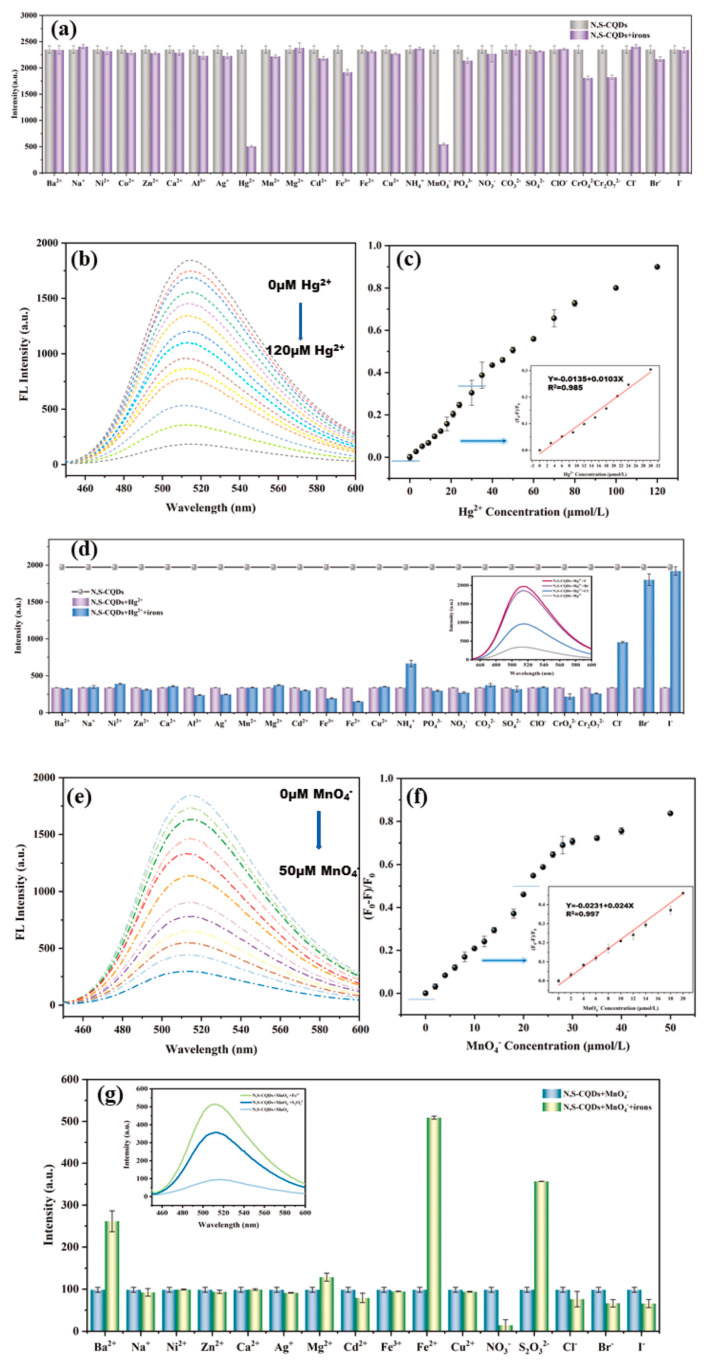
(**a**) Fluorescence intensity of N, S−CQDs solution in presence of different ions, where FL indicates fluorescence; (**b**) fluorescence spectra; (**c**) fluorescence quenching ratio ((F_0_ − F)/F_0_) of N, S−CQDs solution after adding different concentrations of Hg^2+^; (**d**) fluorescence intensity of N, S−CQDs−Hg^2+^ solution after adding different ions; (**e**) fluorescence spectra; (**f**) fluorescence quenching ratio ((F_0_ − F)/F_0_) of N, S−CQDs solution after adding different concentrations of MnO^4−^; (**g**) fluorescence intensity of N, S-CQDs−MnO^4−^ solution after adding different ions [100].

The up-conversion fluorescence offers notable benefits in biomedical applications, such as non-invasiveness, deeper tissue penetration with NIR radiation, and reduced biological interference. Zhang et al. [99] synthesized CDs with both up- and down-conversion PL from coffee beans using a hydrothermal approach, showcasing their utility in imaging Fe^3+^ ions and intracellular sensing, indicative of their potential in bioscience. Similarly, Gao et al. [100] created N, S-doped carbon quantum dots (N, S-CQDs) with a high QY and green fluorescence via hydrothermal synthesis, employing ASDA-Na4 and m-phenylenediamine as the precursors. These N, S-CQDs exhibited exceptional optical attributes, including stability, intense fluorescence, and up-conversion luminescence, proving effective in detecting MnO^4−^ and Hg^2+^ in water, with the advantages of affordability, straightforward visual assessment, and ease of use (Figure 9).

## 4. Bio-Applications

CDs offer significant advantages over conventional organic dyes and semiconductor quantum dots, including robust fluorescence, high photostability, minuscule size, and excellent water solubility. Green CDs stand out for their enhanced biocompatibility and reduced cytotoxicity, making them especially suited for biomedical applications. Their outstanding optical and biological features render green CDs optimal candidates for use in biomedical and biotechnological fields. The upcoming sections delve into the latest advancements in the use of green CDs within these domains, showcasing their potential and innovative applications in various biomedical and biotechnological contexts.

Table 2 summarizes recent articles related to green CDs. The methods used include hydrothermal treatment, microwave radiation, and pyrolysis, as well as the other methods mentioned above. The size of CDs, also described above, is typically less than 10 nm. Their applications are in bioimaging, biosensing, and the detection of metal ions. We further explore their main applications in more detail in the following section.

### 4.1. Bioimaging

The ultrasmall size of CDs makes them promising candidates for bioimaging applications. However, despite their widespread use, there are challenges associated with the specific targeting of structures in biological samples. To address these challenges, various strategies have been developed to achieve the targeted bioimaging of cancer cells. In the early stages, researchers discovered the accumulation effect of CDs in tumor sites and developed uptake-accumulation-targeted imaging techniques. Recently, there has been a growing focus on enhancing the targetability of CDs. With advancements in nanotechnology, CDs have been further developed for tumor-targeted imaging through rational design. Various targeting moieties, such as aptamers, peptides, small molecules, and antibodies, have been explored to boost the internalization of CDs into cells and tissues via ligand–receptor interactions. These targeted methods aim to achieve high specificity in cancer cell targeting and efficient distribution, thereby minimizing the side effects caused by nonspecific binding. More importantly, several studies have observed and confirmed the special targeting markers of CDs in specific tumors, enabling novel applications in self-targeting bioimaging and offering promising approaches for cancer diagnosis. To date, the CDs-related targeted bioimaging oof tumors has shown great promise in the clinical diagnosis of various cancers.

**Figure 10:**
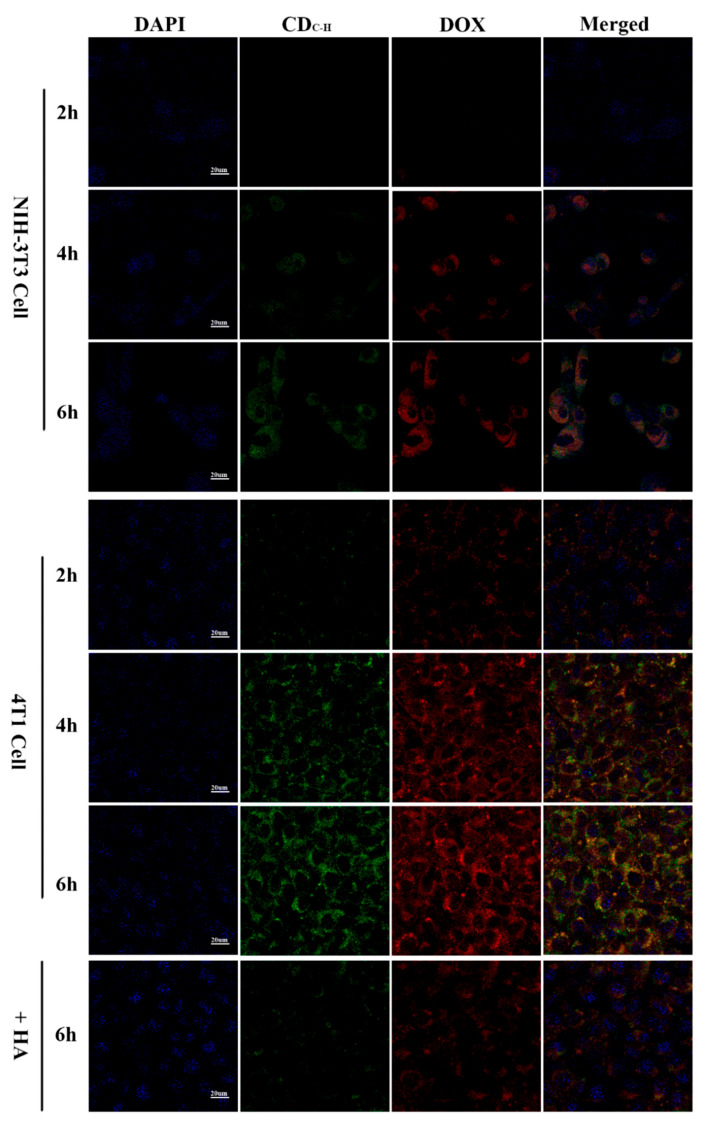
CLSM images of NIH-3T3 and 4T1 cells pretreated with DOX–CDC-H complexes for 2, 4, and 6 h at 37 °C under excitations of 405, 488, and 514 nm, and emissions of 447, 525, and 580 nm, respectively [130].

Wang et al. [130] developed CDs with superior water solubility and stable luminescent properties using hyaluronic acid and carboxymethyl chitosan via a hydrothermal pathway. A targeted, antitumor drug delivery system was formed by loading DOX, a common chemotherapeutic agent, with the CDs. The study results confirmed that the target cell imaging of DOX-CDs was achieved by specifically binding with the CD44 receptors, as shown in Figure 10. These CDs have potential applications in bioimaging and antitumor drug delivery. In a similar vein, Song et al. [125] synthesized peptide-conjugated CDs (TAT-CDs) from formic acid and tryptophan, achieving optimal fluorescent quantum yields conducive to both one- and two-photon nuclear imaging. Notably, the two-photon imaging using TAT-CDs yielded clearer results, particularly in delineating the nuclear details, compared to one-photon imaging. These studies underscore the potential of CDs, especially when conjugated with targeting peptides, for enhancing the specificity and clarity of cellular imaging.

Shen et al. [131,132] introduced a simple one-pot synthesis method for CDs using the hydrothermal carbonization of citric acid and ethylene imine polymer with minor modifications. These CDs were utilized to develop a cancer-targeting and CTSB stimulus-responsive ratio metric nanoprobe, AS_1411_–Ce6–CQDs. Notably, the nanoprobe could integrate a cancer-targeting recognition moiety shift into a single matrix to report CTSB activity specifically, and exhibited an excellent specificity for the ratio metric fluorescent sensing of CTSB activity, demonstrating its potential applications in early cancer diagnosis and precise imaging, and rendering it a unique “turn-on” FL probe for the targeted imaging of cancer cells. Concurrently, Wang et al. [133] crafted CDs from tryptophan and sorbitol via a one-pot hydrothermal pathway. These were specifically designed to monitor tumor cells through target FL imaging. These CDs showcased their potential as a promising anticancer nanotheranostic strategy when integrated into diagnosis, targeting, and therapy.

**Figure 11:**
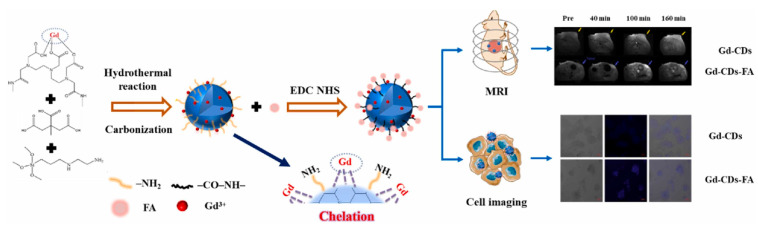
Schematic diagram of hydrothermal synthesis of Gd-CDs as FL-MR probe [123].

Recently, Du et al. [123] synthesized gadolinium-doped CDs (Gd-CDs) via a hydrothermal method using citric acid as the carbon source and gadodiamide as the gadolinium source. Folic acid (FA), which is highly expressed in liver cancer, was then used as the targeting component to modify the Gd-CDs, resulting in a targeted imaging agent (Gd-CDs-FA). The results showed that the Gd-CDs-FA exhibited an excellent FL–magnetic resonance targeting imaging ability for liver cancer, overcoming the limitations of single molecular imaging probes, in terms of sensitivity and soft tissue resolution (Figure 11).

Additionally, Kumar et al. [134] synthesized CDs using a simple hydrothermal method with citric acid and palm/oyster shell waste. The synthesized CDs were evaluated as inflammatory marker dyes in various cell lines, including human dermal fibroblasts, HeLa cells, human-induced pluripotent stem cells, chondrocytes, and cardiomyocytes, and were tested for cytotoxicity. The results showed that the CDs possessed excellent optical properties and biocompatibility, indicating their great potential in bioimaging applications.

### 4.2. Biosensing

In biosensors, CDs enhance the convenience and efficiency of biosensors with their low cytotoxicity and ample surface for functionalization, making them excellent for specific FL-based sensing. The interaction of CDs with metal ions, pivotal in biosensing, is governed by mechanisms including the inner filter effect absorption, photo-excited electron transfer, and FL resonance energy transfer. These interactions facilitate the selective detection of metal ions, which is crucial in environmental monitoring and pollution control. CDs’ inherent oxygen-containing groups, such as hydroxyls and carboxyls, enable strong interactions with metal ions, altering the CDs’ properties and enhancing their sensing capabilities.

**Figure 12:**
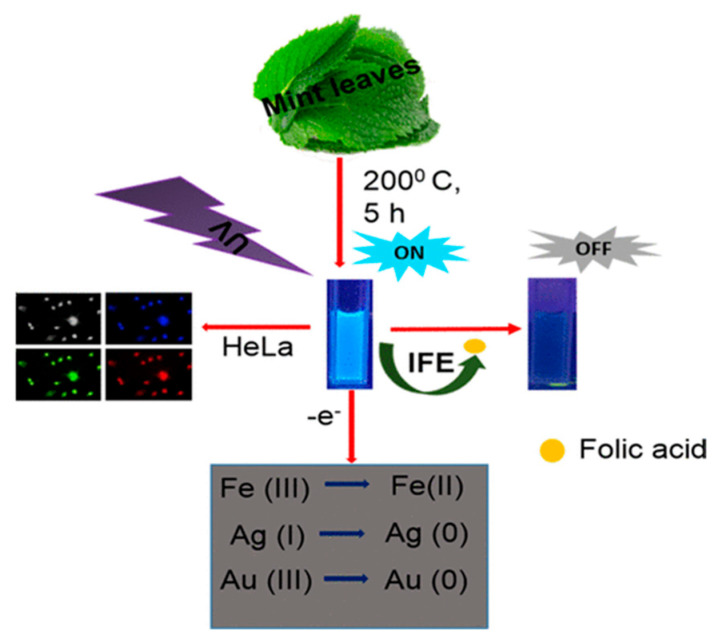
M−CDs utilized as biomarkers, biosensors, and reductants, where IFE indicates inner filter effect [107].

Raveendran et al. [107] used mint leaf extract to synthesize green CDs (M-CDs), showcasing their versatile applications in biomedicine and environmental science. These M-CDs can serve as biomarkers, biosensors, and reductants, demonstrating their multifaceted utility, as shown in Figure 12. Specifically, the authors employed M-CDs for the fluorometric detection of FA, utilizing a quenching mechanism attributed to the inner filter effect. This application underscores M-CDs’ potential in sensitive and selective biosensing, illustrating their promising role in biological and chemical analyses.

Sun et al. [135] developed green CDs using betaine hydrochloride via an eco-friendly and cost-effective calcination method. These CDs demonstrated practical utility in detecting Pb (II) ions, showcasing their potential as effective sensing materials. Furthermore, their anticipated applications include zebrafish and cell imaging, suggesting their broad relevance to biological research and environmental monitoring. Sun et al.’s study highlighted the versatility of G-CDs and their promising future in both sensing and imaging applications.

**Figure 13:**
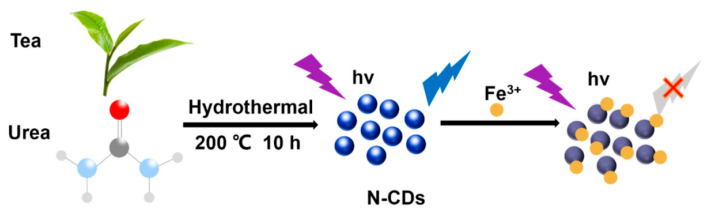
Preparation of N–CDs and applications for Fe^3+^ detection, where the purple symbol indicates the excitation wavelength of 360 nm ; the blue symbol indicates whether the carbon dots have fluorescence properties under the excitation wavelength of 360 nm and hv indicates high volume [136].

**Figure 14:**
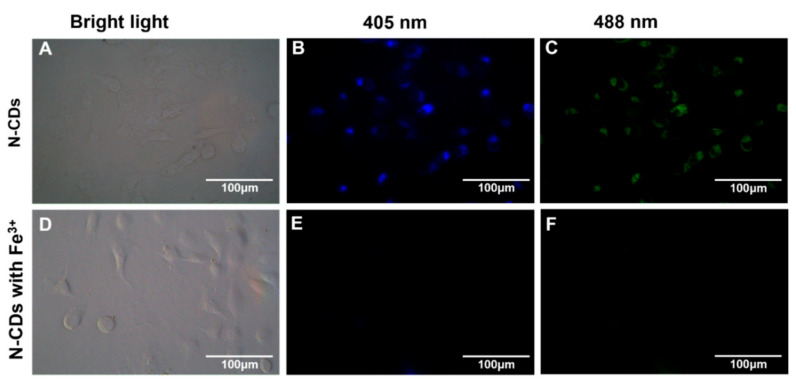
FL microscope images of A549 cells incubated with N-CDs (100 μg·mL^−1^) in the absence (**top**) and presence (**bottom**) of Fe^3+^ under bright-field microscopy (**A**,**D**) and excitations of 405 nm (**B**,**E**) and 488 nm (**C**,**F**).

Ge et al. [136] synthesized nitrogen-doped CDs (N-CDs) from tea leaves using a hydrothermal method, achieving high stability and biocompatibility. These N-CDs were adeptly used to detect Fe^3+^ ions in cells via FL quenching behavior (Figure 13). Additionally, the N-CDs were evaluated as fluorescent probes for the intracellular multicolor imaging and sensing of Fe^3+^, detecting Fe^3+^ at the cellular level (Figure 14). These successful detection applications and positive biocompatibility results underscore the potential of these N-CDs in fields such as iron level detection and cell imaging, highlighting their versatility and applicability in advanced biomedical research.

### 4.3. Drug/Gene Delivery

A diverse array of drug delivery systems have been engineered to enhance the precision of therapeutic agent targeting, with CDs emerging as a promising vehicle, especially their green variant. These green CDs are equipped with various surface functional groups that facilitate the attachment and conveyance of therapeutic agents, whether drugs or genes. They enable this through non-covalent interactions, such as electrostatic forces, hydrogen bonding, and hydrophobic interactions, or through direct covalent bonding. This versatility in binding mechanisms enables green CDs to serve as adaptable and efficient carriers in targeted drug delivery systems, highlighting their potential for advancing therapeutic methodologies.

**Figure 15:**
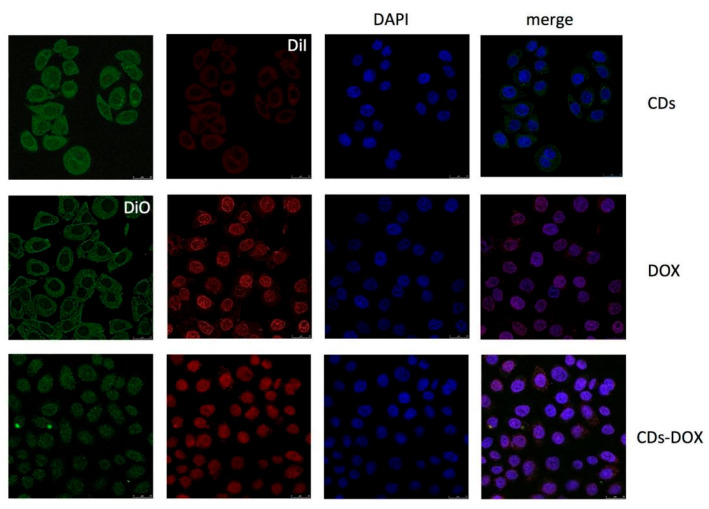
Confocal microscopy images of ACC-2 cells mixed with CDs, DOX, and CDs-DOX for 4 h. The CDs-DOX complexes showed distinct nuclear localization, which was in contrast to the CDs and free DOX [137].

Yuan et al. [137] synthesized green CDs from milk via hydrothermal treatment and conjugated them with DOX, observing enhanced anticancer efficacy. The CD-DOX complexes improved drug localization within tumor cell nuclei and heightened apoptosis in ACC-2 cells compared to DOX alone (Figure 15). In a related study, Wang et al. [32] derived fluorescent CDs from Tsingtao^®^ beer, forming complexes with DOX. These complexes demonstrated sustained cytotoxic effects due to the controlled release of DOX, with internalization confirmed via confocal microscopy. Additionally, Bayda et al. [127] created carbon nanoparticles (CNPs) from black tea, which, when loaded with DOX, circumvented lysosomal capture and were efficiently distributed in the cytoplasm, leading to improved tumor suppression. This was attributed to their better pharmacokinetic properties, establishing CNPs as a versatile and safe option for drug delivery. Together, these studies underscore the potential of CDs and CNPs as innovative carriers in cancer therapy, enhancing drug efficiency and delivery mechanisms.

In their study, Rezaei et al. [138] crafted CDs to serve as a gene delivery vector from chitosan via hydrothermal treatment with arginine as the surface passivation agent. Then, a carboplex was formed via the electrostatic conjugating of arginine–CQDs with DNA to protect it from enzymatic degradation. The arginine–CQDs carboplex demonstrated an excellent gene transfer ability with minimum toxicity compared to the “gold standard” PEI (polyethylenimine) polyplex, with high long-term stability, demonstrating its potential applications as an efficient gene delivery vector. In another study, Ghataty et al. [70] developed highly fluorescent CDs, and the obtained CDs were investigated as fluorescent nano-biocarriers for linezolid drug delivery. The effective wound healing performance of the CDs-mediated delivery system was evaluated through various in vitro and ex vivo assays, illustrating its potential in pharmaceutical applications, as it comprises promising drug delivery nano-biocarriers for effective wound healing applications.

### 4.4. Antimicrobial and Antiviral Effects

The threat posed by viral diseases to human and animal health underscores the crucial need for advanced antiviral research to ensure public safety. In this context, CDs have emerged as promising tools in the antiviral domain. Researchers are actively investigating the potential of new antiviral CDs, aiming to combine effective viral inhibition with superior biocompatibility. By focusing on the development and application of CDs in the antiviral arena, the scientific community is working toward innovative solutions that can offer enhanced protection against viral pathogens, leveraging the unique properties of CDs to advance public health measures.

**Figure 16:**
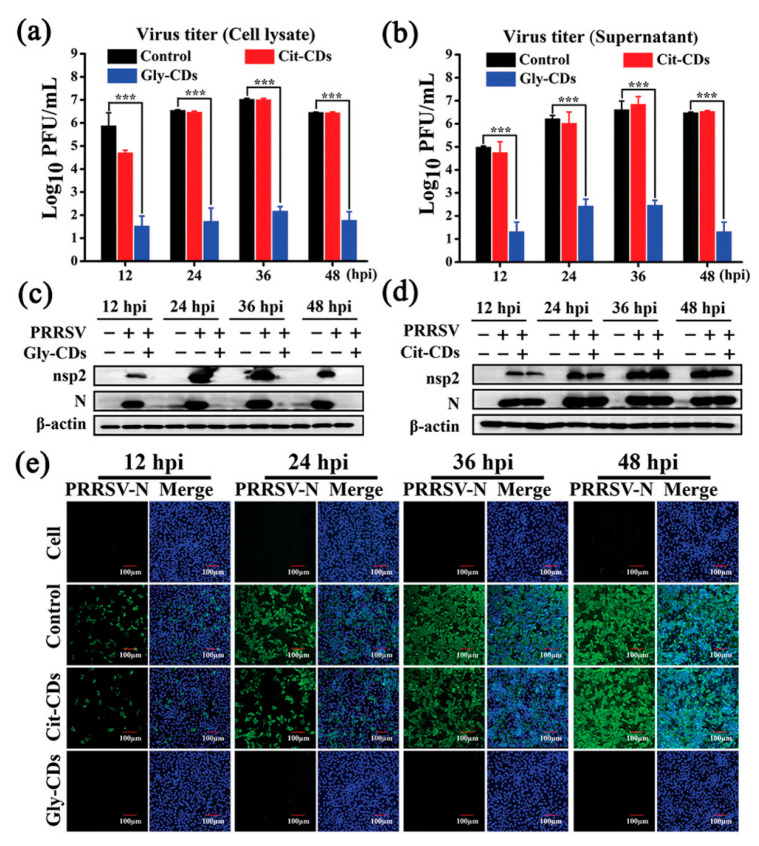
Antiviral activity of Gly CDs on PRRSV. Growth curves of (**a**) intracellular and (**b**) supernatant PRRSV treated with Gly CDs and citric acid-based CDs (Cit CDs) detected via plaque assay; mean value was calculated by *t*-test (mean ± SD, *n* = 3). *** *p* < 0.001, compared with indicated group. Western blot analysis of expression levels of PRRSV (**c**) nonstructural protein 2 and (**d**) N proteins under treatment with Gly CDs and Cit CDs at concentration of 0.30 mg mL^−1^. (**e**) Immunofluorescence assay images of PRRSV-infected MARC-145 cells treated and untreated with 0.30 mg mL^−1^ Gly CDs and Cit CDs at 12, 24, 36, and 48 h post-infection, respectively. Blue represents nucleus and green represents N protein of PRRSV; field of view is random. Scale bar = 100 µm [139].

Tong et al. [139] synthesized biocompatible CDs from glycyrrhizic acid (Gly CDs) using a hydrothermal method, and discovered their potent antiviral properties against porcine reproductive and respiratory syndrome virus (PRRSV). These Gly CDs demonstrated the ability to reduce PRRSV proliferation significantly, diminishing viral titers by up to five orders of magnitude, as shown in Figure 16. Their study further revealed that Gly CDs could thwart PRRSV invasion and replication, enhance antiviral immune responses, and reduce the PRRSV-induced accumulation of intracellular reactive oxygen species (ROS). This research underscores the potential of Gly CDs as an innovative antiviral agent, offering new avenues for combating viral infections effectively.

The rise in antibiotic-resistant microbes presents a formidable challenge to public health, and is exacerbated by the increasing incidence of microbial resistance to conventional antibiotics. In this context of escalating antibiotic resistance, exploring antimicrobial materials and innovative microbial probes is imperative. Recently, antimicrobial nanomaterials have emerged as promising tools with which to combat infectious diseases. Among these, CDs and CQDs stand out because of their nanoscopic size, high biocompatibility, exceptional optical characteristics, and functionalizable surfaces. These properties make CDs/CQDs valuable for microbial imaging, detection, and inactivation, offering new strategies with which to tackle infections and understand microbial behaviors, thus contributing significantly to the field of antimicrobial research.

**Figure 17:**
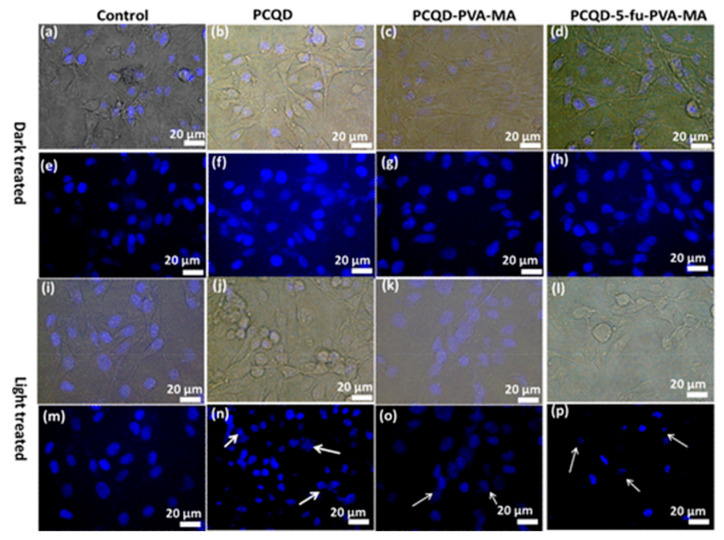
Bright-field and DAPI-stained nuclei of B16F10 melanoma cell lines under dark conditions and laser illumination. (**a**,**e**,**i**,**m**) Control, (**b**,**f**,**j**,**n**) PCQD treated, (**c**,**g**,**k**,**o**) PCQD-PVA-MA treated, and (**d**,**h**,**l**,**p**) PCQD-5Fu-PVA-MA treated. The arrows in subfigure n, o, j point to the nuclei with different degrees of deformation. Obtained from immunofluorescence imaging [104].

Panda et al. [104] obtained CDs-incorporated PVA-MA (polyvinyl alcohol and methacrylic acid) hydrogel from papaya leaf via a hydrothermal method. The obtained CDs-incorporated PVA-MA hydrogel could produce ROS and a photothermal effect under near-infrared (NIR) light, which induced antibacterial activity in both Gram-negative and -positive bacteria. Their study confirmed that this hydrogel design exhibited an antimicrobial effect, demonstrating its potential for improving antimicrobial properties and underscoring its value as a prospective material for the NIR-responsive release of the anticancer drug 5-Fu, offering promising avenues for developing advanced antimicrobial agents (Figure 17).

### 4.5. Formatting of Mathematical Components

Tumors significantly impact human health, and while conventional treatments like surgery, chemotherapy, and radiotherapy are effective, they often come with limitations, such as collateral tissue damage, side effects, and resistance development [140]. Phototherapy, encompassing photodynamic therapy (PDT) and photothermal therapy (PTT), is emerging as a promising alternative, offering minimal invasiveness and high precision. These therapies leverage the light-induced generation of ROS or localized hyperthermia to target and destroy cancer cells while sparing healthy tissue. The advent of nanoparticle-mediated cancer theranostics has further enhanced the potential for precise diagnosis and treatment. In this context, CDs stand out for their excellent biocompatibility, FL, facile surface modification, and efficient synthesis, particularly demonstrating promise in PDT and PTT applications. Thus, CDs are increasingly being recognized for their potential to revolutionize cancer theranostics, providing targeted, efficient, and less invasive treatment options.

**Figure 18:**
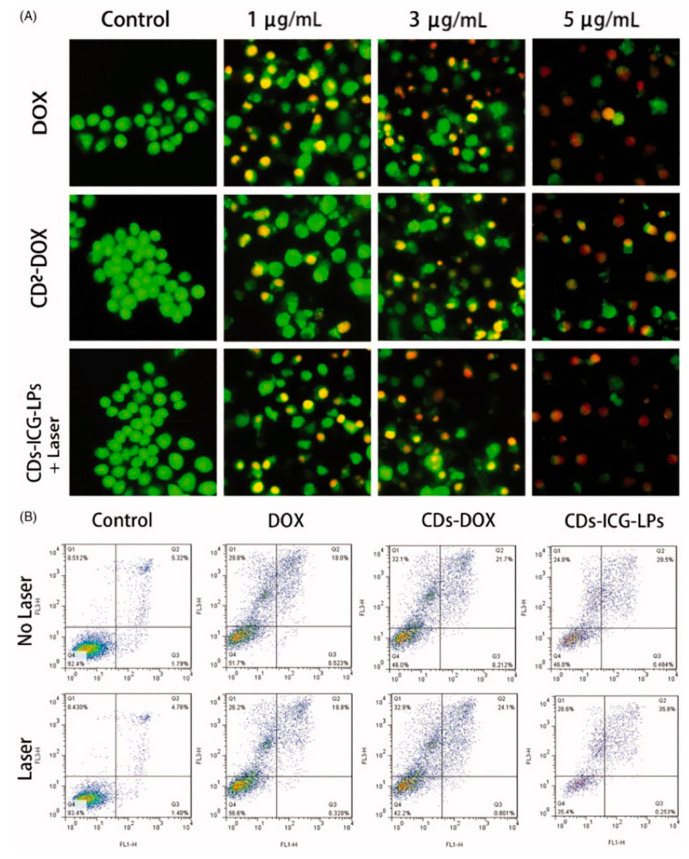
(**A**) Acridine orange/Ethidium bromide staining of apoptotic HepG2 cells incubated with free DOX, CDs-DOX, and laser irradiated CDs-ICG-LPs for 24 h. (**B**) Flow cytometric analysis of apoptotic/necrotic cells in HepG2 cells treated with free DOX, CDs-DOX, and CDs-ICG-LPs for 24 h, untreated cells were used as a control and photothermal was evaluated as well, where the Q1 indicates necrotic cells, Q2 indicates late apoptotic cells, Q3 indicates early apoptotic cells, Q4 indicates intact cells. The flow cytometry data for HepG2 cells treated with CDs-DOX and CDs-ICG-LPs for the referential times. The DOX-CDs displayed higher cellular uptake when compared with the free DOX group. The CDs-ICG-LPs with laser radiation significantly increased the uptake rate of DOX in the HepG2 cells compared to the without-laser group, indicating the enhanced internalization of drugs through the liposome formulation [142].

Xue et al. [141,142] developed an efficient, one-step hydrothermal method to synthesize polyethylene glycol (PEG)-functionalized CDs (CDs@PEG) using citric acid, PEG, and ethylenediamine, exhibiting strong-blue FL. Utilizing a modified reverse-phase evaporation technique, they created DOX-CDs and indocyanine green-loaded liposomes (CDs-ICG-LPs), achieving high drug-loading efficiency. These CDs-ICG-LPs showed superior properties compared to free ICG or DOX, including stable spectral characteristics, FL, size stability, and monodispersity. Upon laser irradiation, these nanocomposites demonstrated accelerated DOX release and an enhanced thermal response, facilitating the synergistic apoptosis and death of HepG2 cells. Furthermore, in vivo studies confirmed their elevated antitumor efficacy against H22 cells and their potential to suppress tumor growth, as shown in Figure 18. Collectively, these findings underscore CDs-ICG-LPs’ potential in chemo-photothermal therapy and targeted cancer imaging, offering a promising avenue for advancing cancer treatment modalities.

Zhou et al. [143] devised a method to synthesize CDs self-assembled with indocyanine green (ICG) dye on bovine serum albumin (BSA), demonstrating their significant potential in tumor photothermal therapy and diagnostic imaging. These CDs-ICG@BSA nanocomposites exhibited strong NIR absorption, which is crucial for effective phototherapy. They were notable for their high photothermal conversion efficiency, enabling efficient light-to-heat energy conversion for targeted tumor ablation. Moreover, these nanocomposites facilitated dual-wavelength bioimaging, allowing for precise tumor localization and the monitoring of the therapeutic process. Their enhanced photothermal and photodynamic activities significantly boosted the therapeutic outcomes of cancer treatment. This innovative approach by Zhou et al. highlights CDs-ICG@BSAs’ potential as multifunctional agents for integrating phototherapy with real-time bioimaging, promising advancements in cancer theranostics and treatment strategies.

### 4.6. Cancer Diagnosis

Recent research trends have emphasized the early detection of cancer using fluorescent materials capable of specific targeting, and CDs have emerged as a focal point because of their exceptional qualities. Their high light stability, low cytotoxicity, superior biocompatibility, and the presence of numerous functional groups on their surface make CDs highly versatile and easily modifiable. These attributes render them invaluable across a spectrum of applications, including photocatalysis, biosensing, bioimaging, drug delivery, and cancer diagnosis, as well as in the development of memory devices. In particular, the ability of CDs to combine diagnostic and therapeutic functions positions them as promising agents in the realm of oncology, where they can contribute significantly to advancements in early cancer detection and treatment.

**Figure 19:**
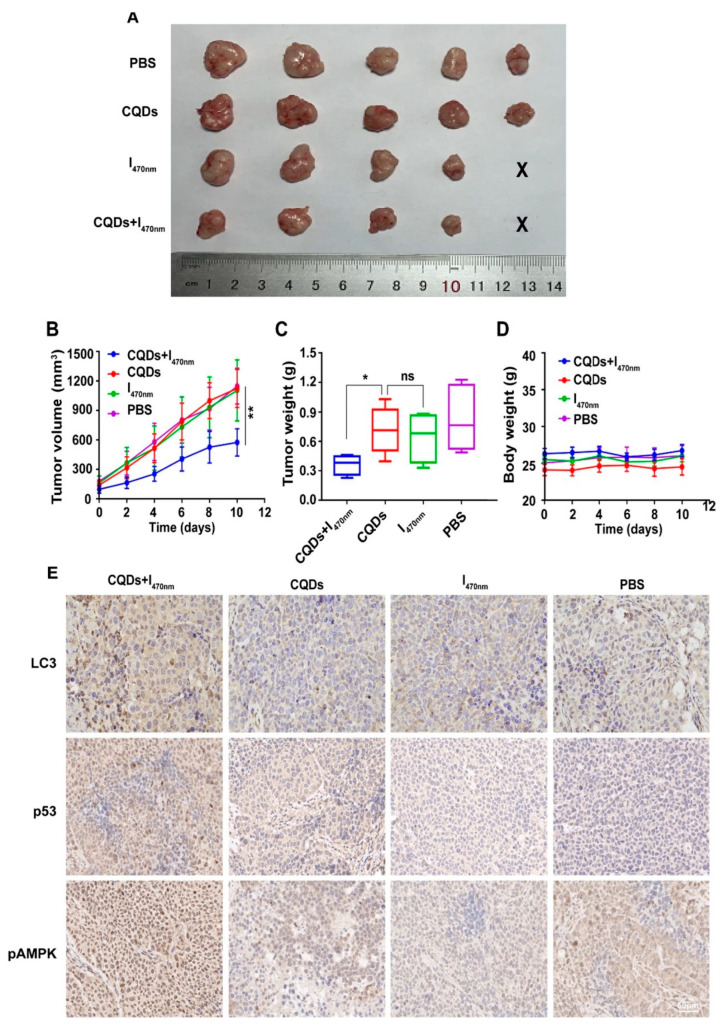
Comparison of the following groups: PBS, CQDs, I_470 nm_, and CQDs  +  I_470 nm_. (**A**) Tumor size (the smallest was in the CQDs  +  I_470 nm_ group); (**B**) tumor volume vs. time; (**C**) tumor weight range (both the tumor volume and the tumor weight in the CQDs  +  I_470 nm_ group showed significant differences compared with the other groups; * *p* < 0.05, ** *p* < 0.01; ns: no significance); (**D**) body weight vs. time, which showed no significant differences between the four groups during the experiment; (**E**) immunohistochemistry, which revealed that LC3, p53, and pAMPK showed the high expression in the CQDs  +  I_470 nm_ group, where LC3, p53, and pAMPK are all proteins that play an important role in autophagy and tumor suppression. CQDs + I_470 nm_: CQDs with 470 nm irradiation; I_470 nm_: 470 nm irradiation without CQDs; CQDs: CQDs; PBS: no CQDs and irradiation [133].

Rashidi et al. [140] synthesized pH-responsive CDs using a hydrothermal method from a green source, achieving blue FL with a high quantum yield. These CDs showed preferential uptake by cancer cells over normal cells, facilitating cancer cell differentiation through FL imaging. This property positions these CDs as valuable tools for early cancer detection and potential targeted therapy applications. Concurrently, Wang et al. [133] produced CDs from tryptophan through a one-pot hydrothermal pathway. These CDs had a much stronger green fluorescence compared to normal hepatocytes, indicating their ability to target hepatocellular carcinoma (HCC) cells. Furthermore, the CDs led to the autophagy of HCC cells by generating ROS. An experiment confirmed that the CDs led to significant tumor inhibition by inducing autophagy with almost no toxicity (Figure 19). The results underscore their potential as a promising anticancer nanotheranostic strategy to be integrated into diagnosis, targeting, and therapy.

Similarly, Yang et al. [141] developed CDs from iron fortifier. The obtained Fe-CDs inhibited tumor growth via intravenous administration until they completely disappeared. Moreover, the Fe-CDs inhibited the epithelial–mesenchymal transition process of tumor cells via the MAPK (mitogen-activated protein kinase)/snail signal pathways, preventing tumor recurrence and metastasis. In addition, the long-term continuous administration of Fe-CDs did not significantly affect the body weight or important organs in mice, indicating its excellent biocompatibility.

The above studies collectively highlight the versatility of CDs synthesized from iron fortifier and how they offer promise as candidates for the next generation of antitumor nanoplatforms.

### 4.7. Pharmaceutical Formulations

The recent surge in CDs applications within nanomedicine underscores their pivotal role, particularly in drug component detection. Their favorable optical attributes, minimal toxicity, nanoscale size, and exceptional biocompatibility make CDs ideal nanoprobes. These properties facilitate sensitive and accurate detection, which is crucial for monitoring drug distribution, release, and interaction at the cellular level. Moreover, CDs’ ease of functionalization allows for targeted delivery and bioimaging, enhancing their utility as diagnostic and therapeutic agents. This burgeoning interest in CDs indicates their potential to revolutionize aspects of drug monitoring, disease diagnosis, and therapeutics, positioning them at the forefront of nanomedical innovation.

**Figure 20:**
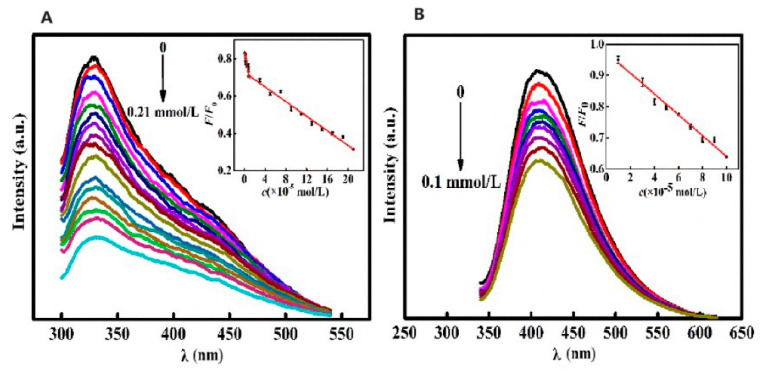
(**A**) FL intensity of CQDs vs. INH concentration under optimal reaction conditions (FL intensity gradually decreased with increase in INH concentration). (**B**) FL intensity of CQDs vs. Fe^3+^ concentration under optimal reaction conditions (FL intensity of CQDs gradually decreased with increase in Fe^3+^ concentration). λ: wavelength. Lines of different color represent a rise in excitation wavelength from 320 nm (top) to 420 nm (down) at intervals of 10 nm [118].

Yu et al. [118] synthesized CQDs from camphor leaves using a hydrothermal method, offering a rapid, simple, and eco-friendly approach. These green-synthesized CQDs served as versatile fluorescent probes for the sensitive and selective detection of isoniazid (INH) and Fe^3+^ without further modifications, as shown in Figure 20. Their study established an FL-based methodology to quantify isoniazid and Fe^3+^ levels, which was effectively applied to analyze isoniazid in pharmaceutical formulations and iron in dietary supplements, yielding satisfactory outcomes.

Elshenawy et al. [142] derived nitrogen and sulfur co-doped CDs (NS-CDs) from glucose and L-cysteine, respectively, employing an eco-friendly and straightforward one-step microwave pyrolysis process that took 90 s. The NS-CDs, notable for their high yield, were utilized as nanoprobes for tilmicosin quantification in milk and pharmaceutical formulations. This was based on a dynamic quenching mechanism, with the NS-CDs demonstrating superior efficacy in tilmicosin detection.

Salman et al. [120] synthesized terbium- and nitrogen-doped CQDs (Tb,N@CQDs), utilizing a microwave-assisted method, from plum juice. The synthesis was characterized by its rapid execution and an impressive QY of 35.44%. The resultant Tb,N@CQDs exhibited an emission peak at 440 nm under an excitation wavelength of 360 nm. Notably, the FL intensity of these CQDs diminished upon the introduction of oxytetracycline (OMC). This method adhered to FDA guidelines, establishing its applicability to clinical research because of its simplicity, cost efficiency, high sensitivity, and exceptional selectivity. Its environmental friendliness was confirmed based on current evaluation indices. This approach was effectively applied to quantify OMC in various matrixes, including milk, human plasma, and pharmaceutical formulations, as well as to facilitate pharmacokinetic studies.

## 5. Conclusions

This review reports the latest progress in CDs technology, detailing their synthesis strategies, optical characteristics, and biomedical uses. CDs synthesis is dichotomized into top–down and bottom–up methodologies, with a substantial emphasis on green synthesis via bottom–up routes, such as hydrothermal treatment, microwave assistance, thermal processing, and extraction. These methods are celebrated for their sustainability, minimal thermal prerequisites, simplicity, cost-effectiveness, and scalability, each of which is meticulously analyzed herein. A diverse gamut of eco-friendly precursors, including fruits, vegetables, spices, non-consumable plants, animal by-products, and recycled substances, are employed in CDs green fabrication. This review also delves into the diverse biomedical applications of CDs, such as bioimaging, biosensing, drug/gene delivery, antimicrobial and antiviral effects, photothermal and PDT, cancer diagnosis, and pharmaceutical formulations.

Biomass resources are often disposed of inefficiently, which causes environmental degradation. These wastes can be turned into bio-products using effective conversion techniques. The synthesis of high-value bio-products from biomass adheres to the principles of a sustainable circular economy in a variety of industries, including agriculture. Recently, fluorescent CDs derived from biowastes have emerged as a breakthrough in the field, showcasing outstanding fluorescence properties and biocompatibility. These CDs exhibit unique quantum confinement properties because of their small size, contributing to their exceptional fluorescence. The significance of their fluorescent properties lies in their versatile applications, particularly in bioimaging and energy devices. Their rapid and straightforward production using green/chemical precursors has further accelerated their adoption in diverse applications. The use of green precursors for CDs not only addresses the biomass disposal issue through a scientific approach, but also establishes a path for a circular economy. This approach minimizes biowaste while harnessing the potential of fluorescent CDs to contribute to sustainable practices in agriculture. This review explores the recent developments and challenges in synthesizing high-quality CDs from agro-residues, shedding light on their crucial role in advancing technologies for a cleaner and more sustainable future.

Looking ahead, we anticipate the emergence of more simple, eco-friendly, efficient, and innovative green synthesis methods, along with the discovery of the enhanced properties and novel applications of these increasingly crucial carbon nanoparticles. For example, with the development of artificial intelligence (AI), we can apply it to the synthesis of CDs [143]. Perhaps we will more efficiently synthesize CDs with better optical properties. Moreover, based on its huge data system, AI may show a better ability to predict, analyze, and identify the priority of CDs. In general, this will provide a new way for the synthesis and application of CDs in the future.

## Figures and Tables

**Table 1:** The size distribution and maximum QY of CDs synthesized by different green methods.

Method	Size Distribution/nm	Maximum QY
Hydrothermal treatment	0.4–18.6	90% [60]
Microwave irradiation	1.7–10	40.38% [79]
Heating	1.4–7	48% [92]
Extraction	2.5–17.73	40% [87]
Pyrolysis	2.26–9.35	20% [93]
Carbonization	1–17	33.7% [94]

**Table 2:** The size, QY, and applications of CDs synthesized using different green sources and methods.

Source	Method	Quantum Yield	Size (nm)	Applications	Ref.
Glucose	Carbonization	nil	~2–10	Cryoprotectants	[101]
Apple seeds	Pyrolysis	20%	<10	Detection of 4-nitrophenol bioimaging	[93]
Chickpea peel	Pyrolysis	10%	7	Bioimaging	[102]
Gynostemma	Calcination	5.7%	2.5	Bioimaging and antioxidants	[103]
Papaya leaf juice	Extraction	12.34%	5–9	Fluorescent antibacterial gel	[104]
Tea leaf residue	Oxidative pyrolysis	14.8%	<10	Sensors for gefitinib	[105]
Amino acids	Hydrothermal	90%	<10	Bioimaging and photocatalysis	[60]
Camel milk	Hydrothermal	24.6%	<10	Mn^7+^ sensing and anti-amyloid and anticancer activities	[42]
Sugarcane bagasse pulp	Hydrothermal	17.98%	0.75–2.75	Nonlinear optical devices, bioimaging, and pharmaceutical applications	[51]
Rice husk	Hydrothermal	3%	4–5	Detection of alcohol vapors	[106]
Mint leaf	Extraction	7.64%	<10	Biosensors, green reductants, and biomarkers	[107]
Chicken eggshell membrane	Hydrothermal	8%	3.35 ± 0.5	Probe for selective DNA recognition	[108]
Citric acid and branched PEI_25000_	Hydrothermal	nil	18.6	Enhanced antibacterial activity	[109]
Passion fruit shells	Hydrothermal	1.8%	<5	Fluorescent probe	[110]
Coriander leaves	Hydrothermal carbonization	6.48%	1.5–3	Detecting Fe^3+^	[36]
Dopamine hydrochloride and o-phenylenediamine	Hydrothermal	1.27%	2.3	Fluorescence probe	[111]
Linseed	Hydrothermal carbonization	14.2%	4–8	Biosensors and bioimaging	[112]
*Bacillus cereus*	Hydrothermal	18.3%	3.3	Detection of p-nitrophenol and bioimaging	[47]
Coffee beans	Hydrothermal	9.8%	3.1–6.0	Fe^3+^ detection and cell imaging	[99]
Carrot juice	Hydrothermal carbonization	5.16%	3–8	Bioimaging	[113]
Date kernels	Hydrothermal	12.5%	1–5	Fluorescence probe and cellular imaging	[114]
Corn flour	Hydrothermal carbonization	7.7%	2–6	Bioimaging and detecting Cu^2+^	[115]
Citric acid	Microwave irradiation	19.2%	10	Detection of Hg^2+^ and I^−^	[116]
ASDA-Na4 and m-phenylenediamine	Hydrothermal	77.68%	0.4–2.6	Detecting Hg^2+^ and MnO^4−^	[100]
Milk	Microwave irradiation	18.7%	<10	Fluorescent labeling, bioimaging, and biosensors	[117]
Camphor leaves	Hydrothermal	nil	1–4	Detecting Fe^3+^ and isoniazid	[118]
Lignocellulose	Microwave irradiation	nil	2–3	Bioimaging	[119]
Plum juice	Microwave irradiation	35.44%	3.1 ± 0.27	Fluorescent nanoprobe	[120]
Gelatin	Microwave irradiation	34%	<10	Biomedical applications	[121]
Mulberry silkworm cocoon	Pyrolysis	6.32%	2.26–9.35	Anti-inflammatory properties	[122]
Gadodiamide	Hydrothermal	48.2%	3.35	Liver cancer-targeted imaging and therapy	[123]
Mangosteen pulp	Heating	nil	5	Cell imaging	[124]
Tryptophan and formic acid	Hydrothermal	58.4%	1.7	Fluorescence imaging	[125]
Honey	Heating	19.8%	2	Imaging and sensing	[82]
Plectranthus amboinicus leaves	Microwave irradiation	17%	2.43 ± 0.02	Fe^3+^ detection and bioimaging	[73]
Coffee beans	Heating	48%	5 ± 2	Nano-sensors and bioimaging agents	[92]
Aloe barbadensis miller	Microwave irradiation	31%	<5	Bioimaging, anticancer, and photocatalytic applications	[77]
Manilkara zapota	Carbonization	7.9%	2.9 ± 0.7	Bioimaging of bacterial and fungal cells	[126]
Microalgae	Carbonization	nil	5	Decoration of TiO_2_ nanoparticles	[74]
Date palm leaf fronds	Carbonization	33.7%	<10	Bioimaging and drug delivery	[94]
Black tea	Carbonization	26%	~17	Drug delivery	[127]
Waste frying oil	Carbonization	3.66%	~2.6	Bioimaging	[128]
Aloe vera leaf gel	Carbonization	16.4%	1.5–3.7	Drug delivery	[129]

## Data Availability

The data presented in this study are available on request from the corresponding author.

## References

[1] Ackermann J., Metternich J.T., Herbertz S., Kruss S. (2022). Biosensing with Fluorescent Carbon Nanotubes. Angew. Chem. Int. Ed..

[2] Ye L., Kollie L., Liu X., Guo W., Ying X., Zhu J., Yang S., Yu M. (2021). Antitumor Activity and Potential Mechanism of Novel Fullerene Derivative Nanoparticles. Molecules.

[3] Srimaneepong V., Skallevold H.E., Khurshid Z., Zafar M.S., Rokaya D., Sapkota J. (2022). Graphene for Antimicrobial and Coating Application. Int. J. Mol. Sci..

[4] Barati F., Avatefi M., Moghadam N.B., Asghari S., Ekrami E., Mahmoudifard M. (2022). A review of graphene quantum dots and their potential biomedical applications. J. Biomater. Appl..

[5] Ajith M.P., Pardhiya S., Rajamani P. (2022). Carbon Dots: An Excellent Fluorescent Probe for Contaminant Sensing and Remediation. Small.

[6] Moreno-Lanceta A., Medrano-Bosch M., Melgar-Lesmes P. (2020). Single-Walled Carbon Nanohorns as Promising Nanotube-Derived Delivery Systems to Treat Cancer. Pharmaceutics.

[7] Mohan H., Bincoletto V., Arpicco S., Giordani S. (2022). Supramolecular Functionalisation of B/N Co-Doped Carbon Nano-Onions for Novel Nanocarrier Systems. Materials.

[8] Chahal S., Macairan J.-R., Yousefi N., Tufenkji N., Naccache R. (2021). Green synthesis of carbon dots and their applications. RSC Adv..

[9] Janus Ł., Piątkowski M., Radwan-Pragłowska J., Bogdał D., Matysek D. (2019). Chitosan-Based Carbon Quantum Dots for Biomedical Applications: Synthesis and Characterization. Nanomaterials.

[10] Jing H., Bardakci F., Akgöl S., Kusat K., Adnan M., Alam M., Gupta R., Sahreen S., Chen Y., Gopinath S. (2023). Green Carbon Dots: Synthesis, Characterization, Properties and Biomedical Applications. J. Funct. Biomater..

[11] Kanwal A., Bibi N., Hyder S., Muhammad A., Ren H., Liu J., Lei Z. (2022). Recent advances in green carbon dots (2015–2022): Synthesis, metal ion sensing, and biological applications. Beilstein J. Nanotechnol..

[12] Mintz K.J., Zhou Y., Leblanc R.M. (2019). Recent development of carbon quantum dots regarding their optical properties, photoluminescence mechanism, and core structure. Nanoscale.

[13] Kar D.K., Praveenkumar V., Si S., Panigrahi H., Mishra S. (2024). Carbon Dots and Their Polymeric Nanocomposites: Insight into Their Synthesis, Photoluminescence Mechanisms, and Recent Trends in Sensing Applications. ACS Omega.

[14] Wang Y., Gu Z., Dong J., Zhu J., Liu C., Li G., Lu M., Han J., Cao S., Chen L. (2024). Green synthesis of chlorella-derived carbon dots and their fluorescence imaging in zebrafish. RSC Adv..

[15] Khan R., Qureshi A., Azhar M., Hassan Z.U., Gul S., Ahmad S. (2024). Recent Progress of Fluorescent Carbon Dots and Graphene Quantum Dots for Biosensors: Synthesis of Solution Methods and their Medical Applications. J. Fluoresc..

[16] Xu X., Ray R., Gu Y., Ploehn H.J., Gearheart L., Raker K., Scrivens W.A. (2004). Electrophoretic analysis and purification of fluorescent single-walled carbon nanotube fragments. J. Am. Chem. Soc..

[17] Sun Y.P., Zhou B., Lin Y., Wang W., Fernando K.A., Pathak P., Meziani M.J., Harruff B.A., Wang X., Wang H. (2006). Quantum-sized carbon dots for bright and colorful photoluminescence. J. Am. Chem. Soc..

[18] Kaczmarek A., Hoffman J., Morgiel J., Mościcki T., Stobiński L., Szymański Z., Małolepszy A. (2021). Luminescent Carbon Dots Synthesized by the Laser Ablation of Graphite in Polyethylenimine and Ethylenediamine. Materials.

[19] Cutroneo M., Silipigni L., Malinsky P., Slepicka P., Franco D., Torrisi L. (2024). Polyvinylalcohol Composite Filled with Carbon Dots Produced by Laser Ablation in Liquids. Polymers.

[20] Kim S., Song Y., Heller M.J. (2017). Seamless aqueous arc discharge process for producing graphitic carbon nanostructures. Carbon.

[21] Zhou X., Zhang Y., Wang C., Wu X., Yang Y., Zheng B., Wu H., Guo S., Zhang J. (2012). Photo-Fenton Reaction of Graphene Oxide: A New Strategy to Prepare Graphene Quantum Dots for DNA Cleavage. ACS NANO.

[22] Li H., Dou Y., Yang H., Xing H., Zhu C., Wang T., Xuan Z., Yang M. (2024). Ce6-modified Fe ions-doped carbon dots as multifunctional nanoplatform for ferroptosis and photodynamic synergistic therapy of melanoma. J. Nanobiotechnol..

[23] Qiao Z.-A., Wang Y., Gao Y., Li H., Dai T., Liu Y., Huo Q. (2010). Commercially activated carbon as the source for producing multicolor photoluminescent carbon dots by chemical oxidation. Chem. Commun..

[24] Magdy G., Belal F., Elmansi H. (2023). Rapid microwave-assisted synthesis of nitrogen-doped carbon quantum dots as fluorescent nanosensors for the spectrofluorimetric determination of palbociclib: Application for cellular imaging and selective probing in living cancer cells. RSC Adv..

[25] Monte-Filho S.S., Andrade S.I.E., Lima M.B., Araujo M.C.U. (2019). Synthesis of highly fluorescent carbon dots from lemon and onion juices for determination of riboflavin in multivitamin/mineral supplements. J. Pharm. Anal..

[26] Laddha H., Yadav P., Sharma M., Agarwal M., Gupta R. (2023). Waste to value transformation: Converting *Carica papaya* seeds into green fluorescent carbon dots for simultaneous selective detection and degradation of tetracycline hydrochloride in water. Environ. Res..

[27] Kailasa S.K., Ha S., Baek S.H., Phan L.M.T., Kim S., Kwak K., Park T.J. (2019). Tuning of carbon dots emission color for sensing of Fe^3+^ ion and bioimaging applications. Mater. Sci. Eng. C Mater. Biol. Appl..

[28] Wang Z., Liao H., Wu H., Wang B., Zhao H., Tan M. (2015). Fluorescent carbon dots from beer for breast cancer cell imaging and drug delivery. Anal. Methods.

[29] Lin R., Cheng S., Tan M. (2022). Green synthesis of fluorescent carbon dots with antibacterial activity and their application in Atlantic mackerel (*Scomber scombrus*) storage. Food Funct..

[30] Zhao X., Liao S., Wang L., Liu Q., Chen X. (2019). Facile green and one-pot synthesis of purple perilla derived carbon quantum dot as a fluorescent sensor for silver ion. Talanta.

[31] Kalkal A., Allawadhi P., Pradhan R., Khurana A., Bharani K.K., Packirisamy G. (2021). Allium sativum derived carbon dots as a potential theranostic agent to combat the COVID-19 crisis. Sens. Int..

[32] Sachdev A., Gopinath P. (2015). Green synthesis of multifunctional carbon dots from coriander leaves and their potential application as antioxidants, sensors and bioimaging agents. Analyst.

[33] Raj S.K., Choudhary B., Yadav A., Patidar R., Mishra A., Kulshrestha V. (2022). Green-synthesized, pH-stable and biocompatible carbon nanosensor for Fe^3+^: An experimental and computational study. Heliyon.

[34] Yu C., Qin D., Jiang X., Zheng X., Deng B. (2021). N-doped carbon quantum dots from osmanthus fragrans as a novel off-on fluorescent nanosensor for highly sensitive detection of quercetin and aluminium ion, and cell imaging. J. Pharm. Biomed. Anal..

[35] Zhang Q., Zhang X., Bao L., Wu Y., Jiang L., Zheng Y., Wang Y., Chen Y. (2019). The Application of Green-Synthesis-Derived Carbon Quantum Dots to Bioimaging and the Analysis of Mercury(II). J. Anal. Methods Chem..

[36] Song Y., Qi N., Li K., Cheng D., Wang D., Li Y. (2022). Green fluorescent nanomaterials for rapid detection of chromium and iron ions: Wool keratin-based carbon quantum dots. RSC Adv..

[37] Cheng Y., Yu G. (2023). Application and Research Status of Long-Wavelength Fluorescent Carbon Dots. Molecules.

[38] Kumar R., Vincy A., Rani K., Jain N., Singh S., Agarwal A., Vankayala R. (2023). Facile Synthesis of Multifunctional Carbon Dots Derived from Camel Milk for Mn^7+^ Sensing and Antiamyloid and Anticancer Activities. ACS Omega.

[39] Dubey P. (2023). An overview on animal/human biomass-derived carbon dots for optical sensing and bioimaging applications. RSC Adv..

[40] Zhang Y., Wang S., Lu F., Zhang M., Kong H., Cheng J., Luo J., Zhao Y., Qu H. (2021). The neuroprotective effect of pretreatment with carbon dots from Crinis Carbonisatus (carbonized human hair) against cerebral ischemia reperfusion injury. J. Nanobiotechnol..

[41] Chatzimitakos T., Kasouni A., Sygellou L., Leonardos I., Troganis A., Stalikas C. (2018). Human fingernails as an intriguing precursor for the synthesis of nitrogen and sulfur-doped carbon dots with strong fluorescent properties: Analytical and bioimaging applications. Sens. Actuators B Chem..

[42] Essner J.B., Laber C.H., Ravula S., Polo-Parada L., Baker G.A. (2016). Pee-dots: Biocompatible fluorescent carbon dots derived from the upcycling of urine. Green Chem..

[43] Zhang S., Zhang D., Ding Y., Hua J., Tang B., Ji X., Zhang Q., Wei Y., Qin K., Li B. (2019). Bacteria-derived fluorescent carbon dots for highly selective detection of p-nitrophenol and bioimaging. Analyst.

[44] Qin K., Zhang D., Ding Y., Zheng X., Xiang Y., Hua J., Zhang Q., Ji X., Li B., Wei Y. (2019). Applications of hydrothermal synthesis of Escherichia coli derived carbon dots in in vitro and in vivo imaging and p-nitrophenol detection. Analyst.

[45] Kousheh S.A., Moradi M., Tajik H., Molaei R. (2020). Preparation of antimicrobial/ultraviolet protective bacterial nanocellulose film with carbon dots synthesized from lactic acid bacteria. Int. J. Biol. Macromol..

[46] Sahoo N.K., Jana G.C., Aktara M.N., Das S., Nayim S., Patra A., Bhattacharjee P., Bhadra K., Hossain M. (2020). Carbon dots derived from lychee waste: Application for Fe(3+) ions sensing in real water and multicolor cell imaging of skin melanoma cells. Mater. Sci. Eng. C Mater. Biol. Appl..

[47] Pandiyan S., Arumugam L., Srirengan S.P., Pitchan R., Sevugan P., Kannan K., Pitchan G., Hegde T.A., Gandhirajan V. (2020). Biocompatible Carbon Quantum Dots Derived from Sugarcane Industrial Wastes for Effective Nonlinear Optical Behavior and Antimicrobial Activity Applications. ACS Omega.

[48] Wang S., Sun W., Yang D.S., Yang F. (2020). Soybean-derived blue photoluminescent carbon dots. Beilstein J. Nanotechnol..

[49] Sun L., Zhang R., Zhang T., Liu X., Zhao Y., Yang M., Cheng H., Zhang Q., Zhang Y., Wu X. (2023). Synthesis, applications and biosafety evaluation of carbon dots derived from herbal medicine. Biomed. Mater..

[50] Fatahi Z., Esfandiari N., Ehtesabi H., Bagheri Z., Tavana H., Ranjbar Z., Latifi H. (2019). Physicochemical and cytotoxicity analysis of green synthesis carbon dots for cell imaging. EXCLI J..

[51] Cong S., Wang N., Wang K., Wu Y., Li D., Song Y., Prakash S., Tan M. (2019). Fluorescent nanoparticles in the popular pizza: Properties, biodistribution and cytotoxicity. Food Funct..

[52] Anpalagan K., Karakkat J.V., Jelinek R., Kadamannil N.N., Zhang T., Cole I., Nurgali K., Yin H., Lai D.T.H. (2023). A Green Synthesis Route to Derive Carbon Quantum Dots for Bioimaging Cancer Cells. Nanomaterials.

[53] Cheng M., Cao L., Guo H., Dong W., Li L. (2022). Green Synthesis of Phosphorescent Carbon Dots for Anticounterfeiting and Information Encryption. Sensors.

[54] Yue J., Li L., Jiang C., Mei Q., Dong W.-F., Yan R. (2021). Riboflavin-based carbon dots with high singlet oxygen generation for photodynamic therapy. J. Mater. Chem. B.

[55] Li S., Wang H., Lu H., Liang X., Wang H., Zhang M., Xia K., Yin Z., Zhang Y., Zhang X. (2021). Sustainable Silk-Derived Multimode Carbon Dots. Small.

[56] Kolanowska A., Dzido G., Krzywiecki M., Tomczyk M.M., Łukowiec D., Ruczka S., Boncel S. (2022). Carbon Quantum Dots from Amino Acids Revisited: Survey of Renewable Precursors toward High Quantum-Yield Blue and Green Fluorescence. ACS Omega.

[57] Ying W., Liu Q., Jin X., Ding G., Liu M., Wang P., Chen S. (2023). Magnetic Carbon Quantum Dots/Iron Oxide Composite Based on Waste Rice Noodle and Iron Oxide Scale: Preparation and Photocatalytic Capability. Nanomaterials.

[58] Jin X.-Y., Ying W.-Y., Che R.-J., Xiao P., Zhou Y.-Q., Liu Y., Liu M.-Y., Chen S.-P. (2022). CQDs/ZnO composites based on waste rice noodles: Preparation and photocatalytic capability. RSC Adv..

[59] Wang X., Feng Y., Dong P., Huang J. (2019). A Mini Review on Carbon Quantum Dots: Preparation, Properties, and Electrocatalytic Application. Front Chem..

[60] Luo K., Wen Y., Kang X. (2022). Halogen-Doped Carbon Dots: Synthesis, Application, and Prospects. Molecules.

[61] Prathap N., Balla P., Shivakumar M.S., Periyasami G., Karuppiah P., Ramasamy K., Venkatesan S. (2023). Prosopis juliflora hydrothermal synthesis of high fluorescent carbon dots and its antibacterial and bioimaging applications. Sci. Rep..

[62] Gholipour A., Rahmani S. (2023). The Green Synthesis of Carbon Quantum Dots through One-step Hydrothermal Approach by Orange Juice for Rapid, and Accurate Detection of Dopamine. J. Fluoresc..

[63] Alam A.-M., Park B.-Y., Ghouri Z.K., Park M., Kim H.-Y. (2015). Synthesis of carbon quantum dots from cabbage with down- and up-conversion photoluminescence properties: Excellent imaging agent for biomedical applications. Green Chem..

[64] Fu X., Fu X., Li W., Chen Y., Cai Z. (2019). Ovalbumin as a Precursor for Green Synthesis of Highly Fluorescent Carbon Dots for Cell Imaging. J. Biomed. Nanotechnol..

[65] Bai X., Ga L., Ai J. (2023). A fluorescent biosensor based on carbon quantum dots and single-stranded DNA for the detection of Escherichia coli. Analyst.

[66] Ghataty D.S., Amer R.I., Amer M.A., Abdel Rahman M.F., Shamma R.N. (2023). Green Synthesis of Highly Fluorescent Carbon Dots from Bovine Serum Albumin for Linezolid Drug Delivery as Potential Wound Healing Biomaterial: Bio-Synergistic Approach, Antibacterial Activity, and In Vitro and Ex Vivo Evaluation. Pharmaceutics.

[67] Muangmora R., Kemacheevakul P., Chuangchote S. (2023). Fiberglass cloth coated by coffee ground waste-derived carbon quantum dots/titanium dioxide composite for removal of caffeine and other pharmaceuticals from water. Heliyon.

[68] Almufarij R.S., Mohamed M.E. (2023). Green Synthesis of a Carbon Quantum Dots-Based Superhydrophobic Membrane for Efficient Oil/Water Separation. Materials.

[69] Architha N., Ragupathi M., Shobana C., Selvankumar T., Kumar P., Lee Y.S., Kalai Selvan R. (2021). Microwave-assisted green synthesis of fluorescent carbon quantum dots from Mexican Mint extract for Fe^3+^ detection and bio-imaging applications. Environ. Res..

[70] Vu Nu T.T., Thi Tran N.H., Truong P.L., Phan B.T., Nguyen Dinh M.T., Dinh V.-P., Phan T.S., Go S., Chang M., Loan Trinh K.T. (2022). Green synthesis of microalgae-based carbon dots for decoration of TiO2 nanoparticles in enhancement of organic dye photodegradation. Environ. Res..

[71] Nkeumaleu A.T., Benetti D., Haddadou I., Di Mare M., Ouellet-Plamondon C.M., Rosei F. (2022). Brewery spent grain derived carbon dots for metal sensing. RSC Adv..

[72] Saini S., Kumar K., Saini P., Mahawar D.K., Rathore K.S., Kumar S., Dandia A., Parewa V. (2022). Sustainable synthesis of biomass-derived carbon quantum dots and their catalytic application for the assessment of α,β-unsaturated compounds. RSC Adv..

[73] Malavika J.P., Shobana C., Ragupathi M., Kumar P., Lee Y.S., Govarthanan M., Selvan R.K. (2021). A sustainable green synthesis of functionalized biocompatible carbon quantum dots from Aloe barbadensis Miller and its multifunctional applications. Environ. Res..

[74] El-Semary M.S., El-Emam A.A., Belal F., El-Masry A.A. (2023). Microwave assisted synthesis of fluorescent hetero atom doped carbon dots for determination of betrixaban with greenness evaluation. RSC Adv..

[75] SalİM F.S., Sargin İ., Arslan G. (2023). Carbon quantum dots and chitosan-based heterogeneous silver catalyst for reduction of nitroaromatic compounds. Turk. J. Chem..

[76] Rawat K.S., Singh V., Sharma C.P., Vyas A., Pandey P., Singh J., Gupta N.M., Sachdev M., Goel A. (2023). Picomolar Detection of Lead Ions (Pb^2+^) by Functionally Modified Fluorescent Carbon Quantum Dots from Watermelon Juice and Their Imaging in Cancer Cells. J. Imaging.

[77] Tohamy H.A.S., El-Sakhawy M., Kamel S. (2022). Eco-friendly Synthesis of Carbon Quantum Dots as an Effective Adsorbent. J. Fluoresc..

[78] Yang X., Zhuo Y., Zhu S., Luo Y., Feng Y., Dou Y. (2014). Novel and green synthesis of high-fluorescent carbon dots originated from honey for sensing and imaging. Biosens. Bioelectron..

[79] Li S., Jiang C., Wang H., Cong S., Tan M. (2018). Fluorescent nanoparticles present in Coca-Cola and Pepsi-Cola: Physiochemical properties, cytotoxicity, biodistribution and digestion studies. Nanotoxicology.

[80] Jiang C., Wu H., Song X., Ma X., Wang J., Tan M. (2014). Presence of photoluminescent carbon dots in Nescafe^®^ original instant coffee: Applications to bioimaging. Talanta.

[81] Liao H., Jiang C., Liu W., Vera J.M., Seni O.D., Demera K., Yu C., Tan M. (2015). Fluorescent Nanoparticles from Several Commercial Beverages: Their Properties and Potential Application for Bioimaging. J. Agric. Food Chem..

[82] Zhang L., Na X., Lai B., Song Y., Wang H., Tan M. (2021). Effects of fluorescent carbon dots from the baked lamb on energy and lipid metabolism. Food Chem..

[83] Wang J., Sahu S., Sonkar S.K., Tackett Ii K.N., Sun K.W., Liu Y., Maimaiti H., Anilkumar P., Sun Y.-P. (2013). Versatility with carbon dots—From overcooked BBQ to brightly fluorescent agents and photocatalysts. RSC Adv..

[84] Cui G., Song Y., Liu K., Tan M. (2021). Interaction of Carbon Dots from Grilled Spanish Mackerel with Human Serum Albumin, γ-Globulin and Fibrinogen. Foods.

[85] Tsai H.-W., Wu T., Hsieh C.-L., Fu S.-F., Wu M.-Y., Lin Y.-W. (2023). Green synthesis of gardenia seeds-based carbon dots for bacterial imaging and antioxidant activity in aqueous and oil samples. RSC Adv..

[86] Atchudan R., Perumal S., Edison T.N.J.I., Sundramoorthy A.K., Vinodh R., Sangaraju S., Kishore S.C., Lee Y.R. (2023). Natural Nitrogen-Doped Carbon Dots Obtained from Hydrothermal Carbonization of Chebulic Myrobalan and Their Sensing Ability toward Heavy Metal Ions. Sensors.

[87] Zhang M., Cheng J., Hu J., Luo J., Zhang Y., Lu F., Kong H., Qu H., Zhao Y. (2021). Green Phellodendri Chinensis Cortex-based carbon dots for ameliorating imiquimod-induced psoriasis-like inflammation in mice. J. Nanobiotechnol..

[88] Vandarkuzhali S.A.A., Jeyalakshmi V., Sivaraman G., Singaravadivel S., Krishnamurthy K.R., Viswanathan B. (2017). Highly fluorescent carbon dots from Pseudo-stem of banana plant: Applications as nanosensor and bio-imaging agents. Sens. Actuators B Chem..

[89] Chatzimarkou A., Chatzimitakos T.G., Kasouni A., Sygellou L., Avgeropoulos A., Stalikas C.D. (2018). Selective FRET-based sensing of 4-nitrophenol and cell imaging capitalizing on the fluorescent properties of carbon nanodots from apple seeds. Sens. Actuators B Chem..

[90] Kavitha T., Kumar S. (2018). Turning date palm fronds into biocompatible mesoporous fluorescent carbon dots. Sci. Rep..

[91] Jeong C.J., Roy A.K., Kim S.H., Lee J.-E., Jeong J.H., In I., Park S.Y. (2014). Fluorescent carbon nanoparticles derived from natural materials of mango fruit for bio-imaging probes. Nanoscale.

[92] Torres F.G., Gonzales K.N., Troncoso O.P., Cañedo V.S. (2023). Carbon Quantum Dots Based on Marine Polysaccharides: Types, Synthesis, and Applications. Mar. Drugs.

[93] Yi H., Liu J., Yao J., Wang R., Shi W., Lu C. (2022). Photoluminescence Mechanism of Carbon Dots: Triggering Multiple Color Emissions through Controlling the Degree of Protonation. Molecules.

[94] Ai L., Yang Y., Wang B., Chang J., Tang Z., Yang B., Lu S. (2021). Insights into photoluminescence mechanisms of carbon dots: Advances and perspectives. Sci. Bull..

[95] Zhang W., Jia L., Guo X., Yang R., Zhang Y., Zhao Z. (2019). Green synthesis of up- and down-conversion photoluminescent carbon dots from coffee beans for Fe^3+^ detection and cell imaging. Analyst.

[96] Gao W., Zhang S., Wang G., Cui J., Lu Y., Rong X., Luo Y., Zhang L., Cheng Z., Gao C. (2023). Nitrogen and sulfur co-doped carbon quantum dots as “on-off-on” fluorescence probes to detect Hg^2+^ and MnO_4_^−^ and improving the photostability of Rhodamine B. Anal. Chim. Acta.

[97] Wang Z., Yang B., Chen Z., Liu D., Jing L., Gao C., Li J., He Z., Wang J. (2020). Bioinspired Cryoprotectants of Glucose-Based Carbon Dots. ACS Appl. Bio Mater..

[98] Singh V., Chatterjee S., Palecha M., Sen P., Ateeq B., Verma V. (2020). Chickpea peel waste as sustainable precursor for synthesis of fluorescent carbon nanotubes for bioimaging application. Carbon Lett..

[99] Wei X., Li L., Liu J., Yu L., Li H., Cheng F., Yi X., He J., Li B. (2019). Green Synthesis of Fluorescent Carbon Dots from Gynostemma for Bioimaging and Antioxidant in Zebrafish. ACS Appl. Mater. Interfaces.

[100] Panda S., ChawPattnayak B., Dash P., Nayak B., Mohapatra S. (2021). Papaya-Derived Carbon-Dot-Loaded Fluorescent Hydrogel for NIR-Stimulated Photochemotherapy and Antibacterial Activity. ACS Appl. Polym. Mater..

[101] Hu Z., Jiao X.-Y., Xu L. (2020). The N,S co-doped carbon dots with excellent luminescent properties from green tea leaf residue and its sensing of gefitinib. Microchem. J..

[102] Thongsai N., Tanawannapong N., Praneerad J., Kladsomboon S., Jaiyong P., Paoprasert P. (2019). Real-time detection of alcohol vapors and volatile organic compounds via optical electronic nose using carbon dots prepared from rice husk and density functional theory calculation. Colloids Surf. A Physicochem. Eng. Asp..

[103] Raveendran V., Kizhakayil R.N. (2021). Fluorescent Carbon Dots as Biosensor, Green Reductant, and Biomarker. ACS Omega.

[104] Pramanik S., Chatterjee S., Suresh Kumar G., Sujatha Devi P. (2018). Egg-shell derived carbon dots for base pair selective DNA binding and recognition. Phys. Chem. Chem. Phys..

[105] Shangguan J., Wu Z., Qiao C., Zhang Y., Li L., Li Q., Gao Y., Yan H., Liu W. (2024). Enhanced Antibacterial Activity against Escherichia coli Based on Cationic Carbon Dots Assembling with 5-Aminolevulinic Acid. ACS Omega.

[106] Yang H., Zhou B., Zhang Y., Liu H., Liu Y., He Y., Xia S. (2020). Valorization of Expired Passion Fruit Shell by Hydrothermal Conversion into Carbon Quantum Dot: Physical and Optical Properties. Waste Biomass Valorization.

[107] Ci Q., Wang Y., Wu B., Coy E., Li J.j., Jiang D., Zhang P., Wang G. (2023). Fe-Doped Carbon Dots as NIR-II Fluorescence Probe for In Vivo Gastric Imaging and pH Detection. Adv. Sci..

[108] Song Y., Yan X., Li Z., Qu L., Zhu C., Ye R., Li S., Du D., Lin Y. (2018). Highly photoluminescent carbon dots derived from linseed and their applications in cellular imaging and sensing. J. Mater. Chem. B.

[109] Liu Y., Liu Y., Park M., Park S.-J., Zhang Y., Akanda M.R., Park B.-Y., Kim H.Y. (2017). Green synthesis of fluorescent carbon dots from carrot juice for in vitro cellular imaging. Carbon Lett..

[110] Amin N., Afkhami A., Hosseinzadeh L., Madrakian T. (2018). Green and cost-effective synthesis of carbon dots from date kernel and their application as a novel switchable fluorescence probe for sensitive assay of Zoledronic acid drug in human serum and cellular imaging. Anal. Chim. Acta.

[111] Wei J., Zhang X., Sheng Y., Shen J., Huang P., Guo S., Pan J., Feng B. (2014). Dual functional carbon dots derived from cornflour via a simple one-pot hydrothermal route. Mater. Lett..

[112] Tabaraki R., Sadeghinejad N. (2018). Microwave assisted synthesis of doped carbon dots and their application as green and simple turn off-on fluorescent sensor for mercury (II) and iodide in environmental samples. Ecotoxicol. Environ. Saf..

[113] Bajpai S.K., D’Souza A., Suhail B. (2019). Blue light-emitting carbon dots (CDs) from a milk protein and their interaction with Spinacia oleracea leaf cells. Int. Nano Lett..

[114] Yu W., Li Q., He L., Zhou R., Liao L., Xue J., Xiao X. (2023). Green synthesis of CQDs for determination of iron and isoniazid in pharmaceutical formulations. Anal. Methods.

[115] Si M., Zhang J., He Y., Yang Z., Yan X., Liu M., Zhuo S., Wang S., Min X., Gao C. (2018). Synchronous and rapid preparation of lignin nanoparticles and carbon quantum dots from natural lignocellulose. Green Chem..

[116] Salman B.I., Hassan A.I., Batakoushy H.A., Saraya R.E., Abdel-Aal M.A.A., Al-Harrasi A., Ibrahim A.E., Hassan Y.F. (2024). Design, Characterization, and Bioanalytical Applications of Green Terbium- and Nitrogen-Doped Carbon Quantum Dots as a Fluorescent Nanoprobe for Omadacycline Analysis. Appl. Spectrosc..

[117] Arsalani N., Nezhad-Mokhtari P., Jabbari E. (2019). Microwave-assisted and one-step synthesis of PEG passivated fluorescent carbon dots from gelatin as an efficient nanocarrier for methotrexate delivery. Artif Cells Nanomed. Biotechnol..

[118] Wang X., Zhang Y., Kong H., Cheng J., Zhang M., Sun Z., Wang S., Liu J., Qu H., Zhao Y. (2019). Novel mulberry silkworm cocoon-derived carbon dots and their anti-inflammatory properties. Artif. Cells Nanomed. Biotechnol..

[119] Du J., Zhou S., Ma Y., Wei Y., Li Q., Huang H., Chen L., Yang Y., Yu S. (2024). Folic acid functionalized gadolinium-doped carbon dots as fluorescence/magnetic resonance imaging contrast agent for targeted imaging of liver cancer. Colloids Surf. B Biointerfaces.

[120] Yang R., Guo X., Jia L., Zhang Y., Zhao Z., Lonshakov F. (2017). Green preparation of carbon dots with mangosteen pulp for the selective detection of Fe^3+^ ions and cell imaging. Appl. Surf. Sci..

[121] Song Y., Li X., Cong S., Zhao H., Tan M. (2019). Nuclear-targeted of TAT peptide-conjugated carbon dots for both one-and two-photon fluorescence imaging. Colloids Surf. B Biointerfaces.

[122] Bhamore J.R., Jha S., Park T.J., Kailasa S.K. (2019). Green synthesis of multi-color emissive carbon dots from Manilkara zapota fruits for bioimaging of bacterial and fungal cells. J. Photochem. Photobiol. B Biol..

[123] Bayda S., Hadla M., Palazzolo S., Kumar V., Caligiuri I., Ambrosi E., Pontoglio E., Agostini M., Tuccinardi T., Benedetti A. (2017). Bottom-up synthesis of carbon nanoparticles with higher doxorubicin efficacy. J. Control. Release Off. J. Control. Release Soc..

[124] Hu Y., Yang J., Tian J., Jia L., Yu J.-S. (2014). Waste frying oil as a precursor for one-step synthesis of sulfur-doped carbon dots with pH-sensitive photoluminescence. Carbon.

[125] Sarkar N., Sahoo G., Das R., Prusty G., Swain S.K. (2017). Carbon quantum dot tailored calcium alginate hydrogel for pH responsive controlled delivery of vancomycin. Eur. J. Pharm. Sci. Off. J. Eur. Fed. Pharm. Sci..

[126] Wang L., Gu D., Su Y., Ji D., Yang Y., Chen K., Pan H., Pan W. (2022). Easy Synthesis and Characterization of Novel Carbon Dots Using the One-Pot Green Method for Cancer Therapy. Pharmaceutics.

[127] Li J.-Y., Liu Y., Shu Q.-W., Liang J.-M., Zhang F., Chen X.-P., Deng X.-Y., Swihart M.T., Tan K.-J. (2017). One-Pot Hydrothermal Synthesis of Carbon Dots with Efficient Up- and Down-Converted Photoluminescence for the Sensitive Detection of Morin in a Dual-Readout Assay. Langmuir.

[128] Shen Y., Wu T., Wang Y., Zhang S.-L., Zhao X., Chen H.-Y., Xu J.-J. (2021). Nucleolin-Targeted Ratiometric Fluorescent Carbon Dots with a Remarkably Large Emission Wavelength Shift for Precise Imaging of Cathepsin B in Living Cancer Cells. Anal. Chem..

[129] Wang Y., Chen J., Tian J., Wang G., Luo W., Huang Z., Huang Y., Li N., Guo M., Fan X. (2022). Tryptophan-sorbitol based carbon quantum dots for theranostics against hepatocellular carcinoma. J. Nanobiotechnol..

[130] Kumar M., Chinnathambi S., Bakhori N., Abu N., Etezadi F., Thangavel V., Packwood D., Sivaniah E., Pandian G.N. (2024). Biomass-derived carbon dots as fluorescent quantum probes to visualize and modulate inflammation. Sci. Rep..

[131] Sun Z., Zhou Y., Zhou W., Luo J., Liu R., Zhang X., Zhou L., Pang Q. (2021). Pb(ii) detection and versatile bio-imaging of green-emitting carbon dots with excellent stability and bright fluorescence. Nanoscale.

[132] Ge G., Li L., Chen M., Wu X., Yang Y., Wang D., Zuo S., Zeng Z., Xiong W., Guo C. (2022). Green Synthesis of Nitrogen–Doped Carbon Dots from Fresh Tea Leaves for Selective Fe^3+^ Ions Detection and Cellular Imaging. Nanomaterials.

[133] Yuan Y., Guo B., Hao L., Liu N., Lin Y., Guo W., Li X., Gu B. (2017). Doxorubicin-loaded environmentally friendly carbon dots as a novel drug delivery system for nucleus targeted cancer therapy. Colloids Surf. B Biointerfaces.

[134] Rezaei A., Hashemi E. (2021). A pseudohomogeneous nanocarrier based on carbon quantum dots decorated with arginine as an efficient gene delivery vehicle. Sci. Rep..

[135] Tong T., Hu H., Zhou J., Deng S., Zhang X., Tang W., Fang L., Xiao S., Liang J. (2020). Glycyrrhizic-Acid-Based Carbon Dots with High Antiviral Activity by Multisite Inhibition Mechanisms. Small.

[136] Yang W.-J., Zhao H.-P., Yu Y., Wang J.-H., Guo L., Liu J.-Y., Pu J., Lv J. (2023). Updates on global epidemiology, risk and prognostic factors of gastric cancer. World J. Gastroenterol..

[137] Yang L., Jiang W., Qiu L., Jiang X., Zuo D., Wang D., Yang L. (2015). One pot synthesis of highly luminescent polyethylene glycol anchored carbon dots functionalized with a nuclear localization signal peptide for cell nucleus imaging. Nanoscale.

[138] Xue X., Fang T., Yin L., Jiang J., He Y., Dai Y., Wang D. (2018). Multistage delivery of CDs-DOX/ICG-loaded liposome for highly penetration and effective chemo-photothermal combination therapy. Drug Deliv..

[139] Zhou X., Cai Q., Zhao S., Ling F., Xiang G., Li L., Wang Y., Li Y., Tang X. (2024). CDs-ICG@BSA nanoparticles for excellent phototherapy and in situ bioimaging. Talanta.

[140] Rashidi E., Esfandiari N., Ranjbar Z., Alvandi N., Fatahi Z. (2021). Designing of a pH-activatable carbon dots as a luminescent nanoprobe for recognizing folate receptor-positive cancer cells. Nanotechnology.

[141] Yang M., Li H., Liu X., Huang L., Zhang B., Liu K., Xie W., Cui J., Li D., Lu L. (2023). Fe-doped carbon dots: A novel biocompatible nanoplatform for multi-level cancer therapy. J. Nanobiotechnol..

[142] Elshenawy E.A., El-Malla S.F., Hammad S.F., Mansour F.R. (2023). Green microwave-prepared N and S Co-doped carbon dots as a new fluorescent nano-probe for tilmicosin detection. Talanta.

[143] Singh A.V., Maharjan R.-S., Kanase A., Siewert K., Rosenkranz D., Singh R., Laux P., Luch A. (2020). Machine-Learning-Based Approach to Decode the Influence of Nanomaterial Properties on Their Interaction with Cells. ACS Appl. Mater. Interfaces.

